# Identifying heat shock-responsive lncRNAs in wheat during grain filling

**DOI:** 10.1186/s12870-026-09785-z

**Published:** 2026-08-25

**Authors:** Weishuang Zhao, Huiqiang Wang, Qiang Li, Ziyue Geng, Jiachang Li, Mengjia Wang, Jiaoteng Bai, Wenchen Qiao, Xigang Liu, Shuzhi Zheng

**Affiliations:** 1 Ministry of Education Key Laboratory of Molecular and Cellular Biology, Hebei Collaboration Innovation Center for Cell Signaling and Environmental Adaptation, Hebei Key Laboratory of Molecular and Cellular Biology, College of Life Sciences, Hebei Normal University, Shijiazhuang, 050024 China; 2 Dry-Land Farming Institute, Hebei Academy of Agricultural and Forestry Science/Key Laboratory of Crop Drought Tolerance Research of Hebei Province, Hengshui, 053000 China

**Keywords:** *Triticum aestivum*, Heat stress response, Long non-coding RNA, Grain-filling stage, WGCNA

## Abstract

**Background:**

Heat stress (HS) during the grain-filling stage severely threatens wheat yield. While long non-coding RNAs (lncRNAs) are known to regulate plant stress responses, their specific roles in wheat grain filling under HS remain poorly understood.

**Results:**

In this study, we integrated population-scale transcriptomics to explore lncRNA-associated thermotolerance networks. Analysis of 41 diverse wheat genotypes under control and HS conditions identified 9,463 high-confidence lncRNA loci and thousands of differentially expressed transcripts. Weighted gene co-expression network analysis (WGCNA) uncovered modules closely linked to thousand-grain weight (TGW), highlighting 79 hub lncRNAs. Association analysis revealed 22 lncRNAs with haplotypes significantly correlated with TGW. Field phenotyping demonstrated that under HS, wheat genotypes the high expression of *MSTRG.64082* exhibited significantly higher TGW than those with medium or low expression. A positive correlation between TGW and the expression level of *MSTRG.64082* was also observed in the 2025 field trials. Furthermore, the heat-induced expression of *MSTRG.64082* was accompanied by the specific upregulation of key heat-responsive genes. Genetic analysis indicated that *MSTRG.64082* harbors a favorable haplotype (HapC) associated precisely with enhanced TGW under HS. Notably the HapC allele displays a temperature-associated latitudinal cline, with its frequency increasing significantly in warmer regions. These findings suggest that *MSTRG.64082* potentially acts as a positive regulator of heat tolerance and may have been subjected to selection pressure in warm climates.

**Conclusions:**

This study provides a comprehensive population-level atlas of heat-responsive lncRNAs during wheat grain filling and highlights *MSTRG.64082* as a promising candidate locus for breeding heat-tolerant wheat varieties.

**Supplementary Information:**

The online version contains supplementary material available at https://doi.org/10.1186/s12870-026-09785-z.

## Introduction

Rising global temperatures, a hallmark of climate change, significantly affect plant growth, development, and distribution. As a major global food crop, bread wheat (*Triticum aestivum* L.), exhibits heightened sensitivity to environmental fluctuations. The grain-filling stage, which is critical for dry matter accumulation and ultimately determines grain weight, is particularly vulnerable to heat stress (HS). Such stress not only compromises grain yield and quality but also accelerates reproductive development, thereby shortening the grain-filling period and reducing thousand-grain weight (TGW) by 10–40% [1–3].

Deciphering the mechanisms of heat tolerance during grain filling is therefore essential for stabilizing wheat yields under global warming. However, current research at this critical stage has predominantly focused on physiological and metabolic phenotypes, with transcriptomic studies largely confined to protein-coding genes. Several key regulators have been identified, for instance, the loss of heat shock transcription factor A1 (TaHSFA1) not only confers heat susceptibility in seedlings but also markedly reduces TGW under HS conditions [4]. Similarly, the heat shock factor TaHSFC2a is highly expressed in filling grains and interacts with TaHSFA2h to activate the transcription of protective genes, including *TaHSP70d* and *TaHSP26* [5, 6]. Beyond heat transcription factors, the heat stress tolerance (HST) locus, *TaHST2* has been finely mapped. It enhances thermotolerance across developmental stages by promoting the conversion of soluble sugars to starch [7]. Concomitantly, metabolic adaptations have been extensively characterized. HS truncates the filling period and reduces its rate, disproportionately impairing starch synthesis relative to protein accumulation. This inhibition targets key enzymes—including ADP-glucose pyrophosphorylase (AGPase) and soluble starch synthase (SSS)—leading to severe defects in starch biosynthesis [8–10], altered granule morphology, and reduced grain size [11]. Consequently, large-scale screening for regulatory factors remains essential for identifying valuable genetic resources to breed heat-resilient wheat varieties.

AS an allopolyploid crop, wheat possesses a vast and complex genome replete with non-coding sequences that harbor critical regulatory elements essential for precise gene expression control [12]. Recent advances have established long non-coding RNAs (lncRNAs) as pivotal regulators of plant growth, development, and stress adaptation [13]. Nevertheless, their specific involvement in HS responses during grain filling remains largely uncharacterized.

In plants, non-coding RNAs (ncRNAs)—primarily including microRNAs (miRNAs), short interfering RNAs (siRNAs), and lncRNAs—function as essential regulatory factors [14]. LncRNAs are defined as functional RNAs exceeding 200 nucleotides with negligible protein-coding potential [15]. Unlike protein-coding genes, lncRNAs employ remarkably diverse regulatory modalities. They can act as guides or scaffolds to recruit chromatin-modifying complexes, such as the Polycomb Repressive Complex 2 (PRC2), to mediate epigenetic silencing via H3K27me3 [16]. Conversely, they can recruit activating complexes like the COMMPASS methyltransferase (via WDR5a) to elevate H3K4me3 levels [17]. Furthermore, lncRNA transcription itself can interfere with neighboring genes by competing for RNA polymerase II [18]. At the post-transcriptional level, lncRNAs function as auxiliary spliceosome components to modulate alternative splicing [19], or serve as endogenous target mimics (eTMs) that sequester miRNAs, thereby alleviating the repression of target genes [20–24]. Collectively, lncRNAs represent versatile regulators that fine-tune diverse biological processes through multilayered transcriptional and post-transcriptional mechanisms.

Accumulating evidence establishes that lncRNAs orchestrate plant growth, development, agronomic traits, and stress resilience. During development, Arabidopsis lncRNAs govern root architecture via auxin signaling [25], photoperiodic flowering [26], blue light morphogenesis [27], and vernalization [17, 28, 29], while also fine-tuning leaf morphology [16, 30]. Regarding agronomic traits, rice lncRNAs, such as *LAIR*, *Ef-cd* and *MIS-SHAPEN ENDOSPERM* (*MISSEN*), modulate grain yield by regulating seed development [31–33]. Specifically, *LDMAR* safeguards pollen fertility under long days [34], while *VIVIpary* controls seed dormancy to prevent pre-harvest sprouting [35]. Despite these advances, the functions of lncRNAs during the grain-filling stage remain largely unexplored.

In stress responses, lncRNAs play multilayered regulatory roles. In rice, *ALEX1* enhances bacterial blight resistance via jasmonic acid biosynthesis [36]. In Arabidopsis, *DRIR* and *DANA1/2* bolster drought tolerance by potentiating abscisic acid (ABA) signaling and hydraulic conductivity [37–39]. The cold-responsive *SVALKA-asCBF1* competitively binds RNA polymerase II to fine-tune cold adaptation [18]. Concerning thermotolerance, pear *HILinc1* interacts with HSFA1b to amplify HS responses [40], while cucumber lncRNAs coordinate defenses through phytohormone pathways and miR9748 interactions [41]. Although comparative transcriptomics has identified candidate thermo-responsive lncRNAs in rice [42], similar research in wheat lags significantly behind.

The completion of the wheat genome sequence has accelerated the identification of lncRNAs, and their molecular mechanisms are gradually being elucidated. Notably, the lncRNA *VAS*—generated via alternative splicing of the *TaVRN1* gene—is relatively well characterized. Expressed during early vernalization, *VAS* interacts with TaRF2b to facilitate the recruitment of the TaRF2b–TaRF2a complex to the *TaVRN1* promoter, thereby activating its expression and accelerating flowering [29]. Functional insights into other wheat lncRNAs have largely emerged from mutant analyses and overexpression studies. For instance, *TalncR9* overexpression enhances drought tolerance [43], whereas *TraesLNC1D26001.1* overexpression impairs seed germination compared to the wild-type cultivar Fielder [44]. Concurrently, bioinformatics approaches have identified lncRNAs associated with various developmental stages and stress responses. These include *TCONS_00130663*, which negatively regulates starch branching enzyme IIb to mediate resistant starch biosynthesis [45], and regulatory networks identified in winter wheat cultivar Dongmai 1 under cold stress [46]. Similarly, analysis of four wheat genotypes during pollen development under HS revealed lncRNA–miRNA–mRNA modules implicated in thermotolerance [47]. However, no comparable studies have focused on the grain-filling stage. Consequently, identifying lncRNAs specifically responsive to HS during this critical yield-forming phase holds significant promise for improving wheat heat tolerance.

In this study, we integrated population-scale transcriptomics to construct the first high-resolution atlas of heat-responsive lncRNAs during wheat grain filling. By analyzing 41 diverse wheat genotypes under control and HS conditions, we identified 9,463 high-confidence lncRNA loci, which exhibited distinct genotype-specific expression patterns compared to mRNAs. WGCNA delineated key modules strongly correlated with TGW, highlighting 79 hub lncRNAs. Subsequent genotype–phenotype association analysis pinpointed 22 lncRNA loci harboring haplotypes significantly associated with TGW. Notably, we identified a heat-induced lncRNA, *MSTRG.64082*, which contains a specific haplotype (HapC) associated with enhanced thermotolerance. The haplotype significantly correlated with increased TGW under HS without compromising yield under normal conditions. Collectively, our work not only reveals a comprehensive lncRNA regulatory landscape in response to HS during grain filling but also provides a promising candidate locus and a functional marker for the molecular breeding of heat-resilient wheat varieties.

## Results

### Identification of HS-responsive lncRNAs during grain filling

**Fig. 1:**
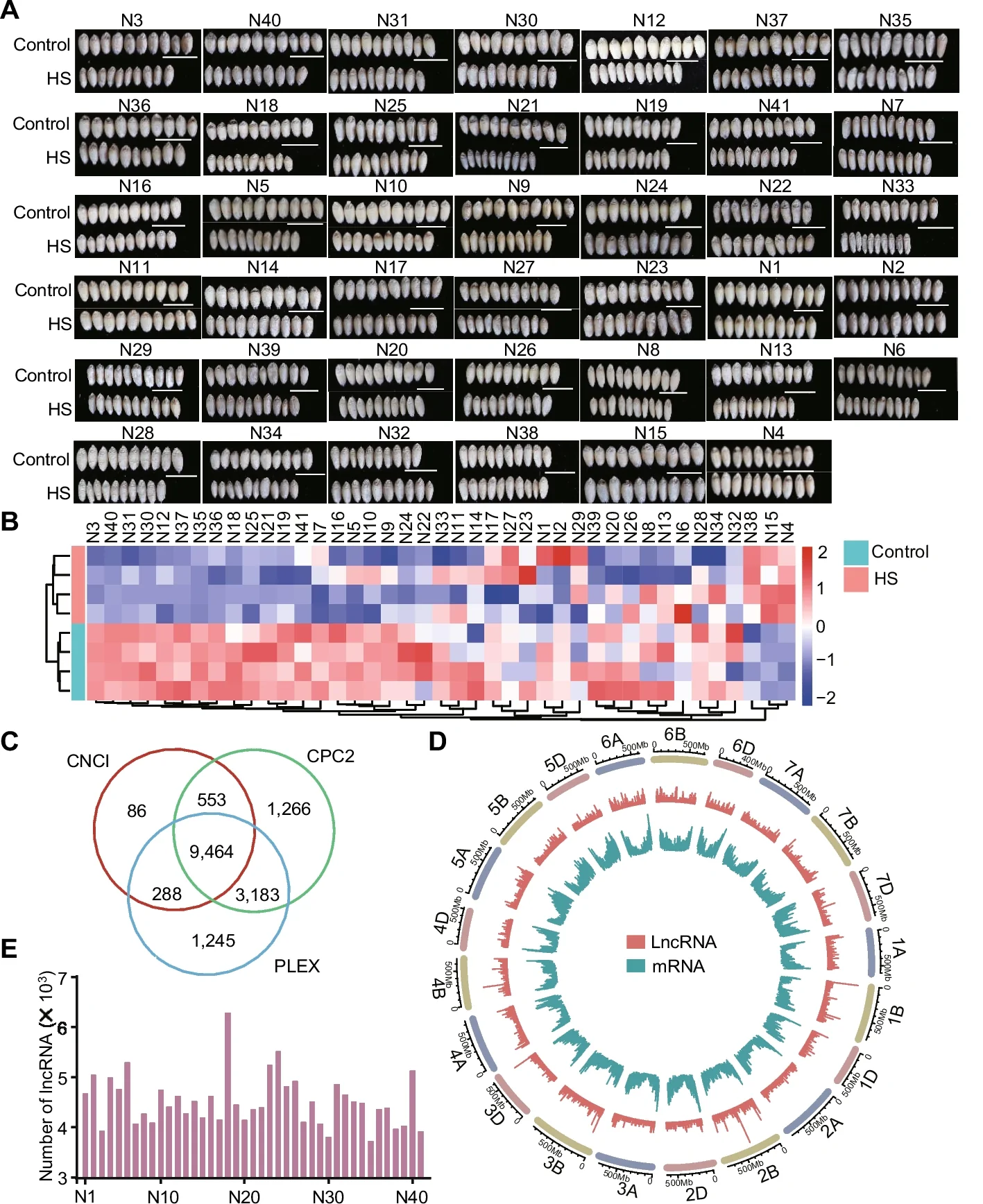
Identification of HS-responsive lncRNAs in wheat during grain filling. **A** Visual comparison of grain morphology from 41 wheat genotypes (N1–N41) cultivated under control and heat stress (HS) conditions. Detailed genetic and agronomic profiles for these 41 genotypes are provided in Table S1. **B** Heatmap displaying thousand-grain weight (TGW) of the 41 wheat genotypes under control and HS conditions. **C** Overlap of identified lncRNA candidates during wheat grain filling under HS, based on Coding Potential Calculator 2 (CPC2), Coding-Noncoding Index (CNCI), and Plant LncRNA Explorer (PLEX) computational pipelines. A high-confidence set of 9,464 lncRNAs (represented in the central intersection) was consistently identified by all three classification tools. **D** Comparative genomic distribution of lncRNAs and mRNAs across wheat chromosomes. Bar lengths represent the cumulative number of identified lncRNAs (orange) and mRNAs (blue) mapped to each chromosome. **E** Abundance of high-confidence lncRNAs annotated within each of the 41 wheat genotypes (N1– N41). Vertical bar indicate lncRNA counts, ranging from approximately 4.0 × 10^3^ to 6.4 × 10^3^ among the genotypes

To investigate the impact of heat stress (HS) on wheat during grain filling, we first examined the elite variety KN9204 under controlled conditions. Exposed to 42 °C from 21 days post-anthesis until seed maturity, significantly impaired the grain-filling process, leading to a marked reduction in seed dry weight compared to controls (Fig. S1). Building on this observation, we expanded our analysis to a broader context using 41 genetically diverse genotypes (Table S1). These were selected as representative accessions from a diversity panel previously utilized in a wheat drought adaptation study [48], capturing a wide spectrum of genetic variation and clustering into three distinct subpopulations based on our RNA-seq data (Fig. S2). To minimize confounding effects from this underlying population structure, we employed a paired comparison design, assessing thousand-grain weight (TGW) via field experiments in Hengshui under both HS and control conditions. Phenotypic responses varied dramatically among genotypes: twelve genotypes (e.g., N3, N40, and N31) displayed significantly lower TGW under HS, indicative of high HS sensitivity, whereas three genotypes (N38, N15, and N4) exhibited increased TGW relative to controls, highlighting distinct HS tolerance (Fig. 1A, B).

To systematically identify lncRNAs in developing grains under HS, we performed integrative analyses on 82 RNA-seq datasets from 41 genotypes. Samples were collected from grains before and after a 3-day HS treatment (42°C) during the grain-filling stage. Sequencing yielded 80 Gb of high-quality clean reads, with an average alignment rate of > 90% to the wheat reference genome (KN9204) (Table S2). Genome alignment and redundancy removal resulted in 424,196 unique transcripts from 241,346 gene loci, including 187,437 protein‑coding transcripts. We then sequentially filtered out transcripts overlapping with protein-coding genes or known non-coding RNAs (405,691), transcripts shorter than 200 nt (41), and those exhibiting coding potential (9,000). As a result, 9,464 transcripts derived from 9,263 distinct loci were retained as high-confidence lncRNA candidates (Fig. 1C, D; Fig. S3; Table S3). The number of identified lncRNAs per cultivar ranged from 3,740 to 6,298 (Fig. 1E).

These lncRNAs exhibited sequence and expression feature distinct from those of mRNAs. Structurally, lncRNAs contained fewer exons, with 89% classified as single‑exon transcripts (Fig. S4A), and possessed significantly shorter transcript lengths (Fig. S4B). Under both control and HS conditions, the median expression abundance of mRNAs was substantially higher than that of lncRNAs (Fig. S4C). Notably, lncRNAs were detected in fewer genotypes on average (8 under control, 3 under HS) compared with mRNAs (24 and 21, respectively) (Fig. S4D), suggesting a stronger genotype-specific expression pattern. This restricted expression profile provided a valuable reference threshold for the subsequent identification of differentially expressed transcripts.

### Pan-transcriptome profiling of HS responses

**Fig. 2:**
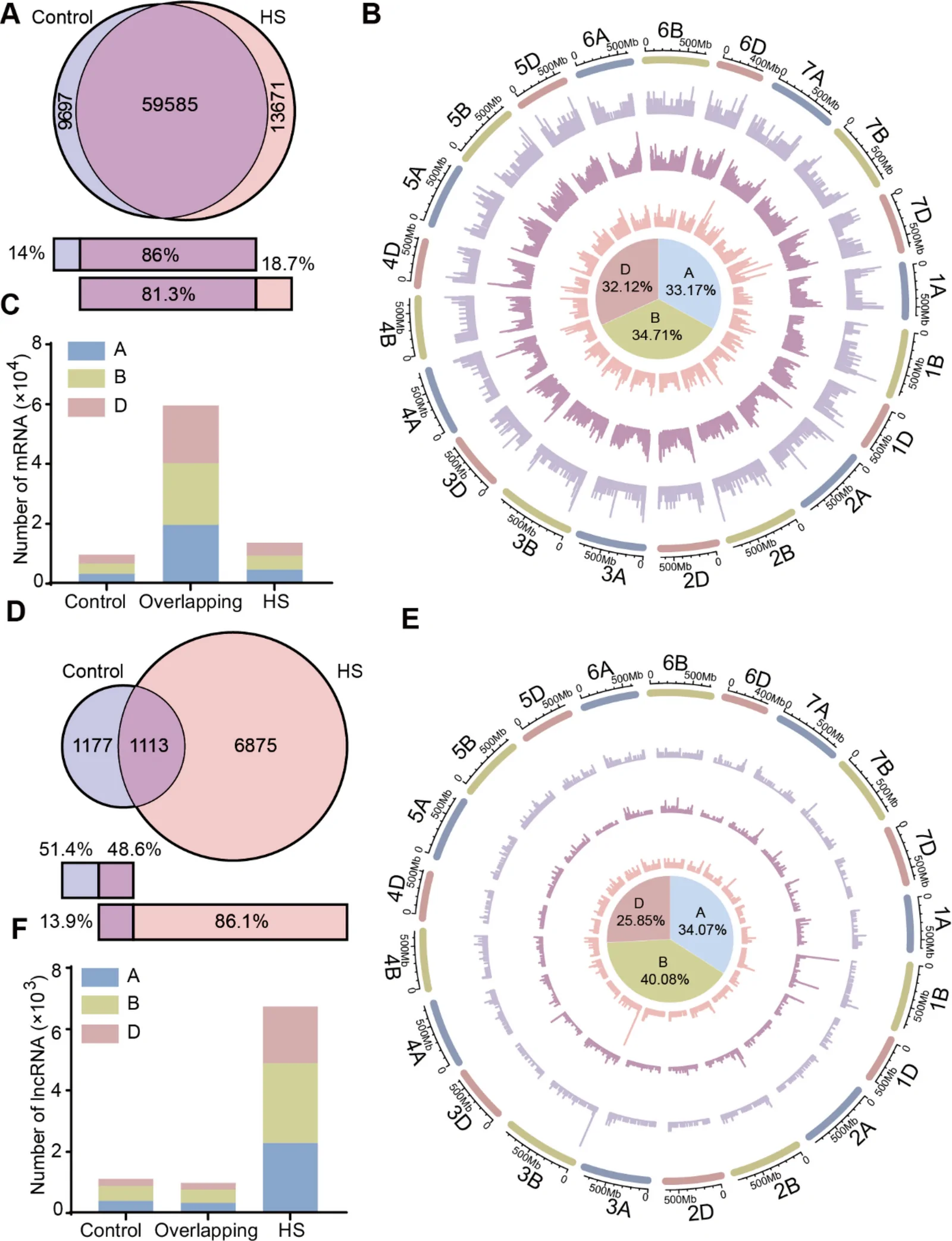
Genome-wide comparative expression landscapes of mRNAs and lncRNAs under control and HS conditions during wheat grain filling. **A** Venn diagram showing the number of mRNAs expressed specifically under control (9697) and HS (13,671) conditions, alongside constitutively expressed mRNAs (59,585) shared between both conditions. **B** Quantitative distribution of expressed mRNAs across the A, B, and D subgenomes of wheat under control and HS conditions. **C** Circos plot illustrating the chromosomal distribution of hot-spot regions for mRNA expression. From the innermost to outermost tracks, concentric rings represent the genomic density of mRNAs uniquely expressed under HS conditions, those expressed under both conditions, and those uniquely expressed under control conditions, respectively. The central pie chart illustrates the genomic distribution of all expressed mRNAs across the A, B, and D subgenomes. **D** Venn diagram depicting the shared (1,113) and specifically expressed lncRNAs under control (1,177) and HS (6,875) conditions. **E** Quantitative distribution of expressed lncRNAs across the A, B, and D subgenomes of wheat under control and HS conditions. **F** Circos plot representing the chromosomal density of expressed lncRNA. The tracks (innermost to outermost) correspond to lncRNAs specific to HS, shared between conditions, and specific to control conditions. The central pie chart depicts the overall distribution of identified lncRNAs across the three subgenomes

To systematically elucidate transcriptional reprogramming during grain filling under HS, we conducted a pan-transcriptome analysis of mRNAs and lncRNAs across 41 wheat genotypes. Alignment of high-quality reads to the wheat reference genome (KN9204) detected 82,953 protein-coding genes and 9,165 lncRNA-expressing loci (Fig. 2A, D). These two transcript classes exhibited distinct expression profiles and genomic distributions. While most mRNAs were constitutively expressed under both conditions, over 70% of lncRNAs were specifically induced by HS (Fig. 2A, D), suggesting that lncRNAs are highly environmentally responsive and likely participate in thermoadaptation.

Genomically, mRNAs were evenly distributed across the A (33.17%), B (34.71%), and D (32.12%) subgenomes (Fig. 2B). In contrast, lncRNAs showed a biased distribution, with the highest proportion in the B subgenomes (40.08%), followed by A (34.07%) and D (25.85%) subgenomes (Fig. 2E). However, the responsiveness to HS did not differ significantly among subgenomes for either transcript type (Fig. 2C, F).

Given the strong genotype-specific expression of lncRNAs (Fig. S4D), distinct thresholds were applied to identify differentially expressed protein-coding genes (DEGs) and differentially expressed lncRNAs (DELs). For protein-coding genes, a transcript was classified as a DEG if it met the criteria of |log₂(fold change)|≥ 1 and showed differential expression in > 36.5% (15/41) of the genotypes. This stringent filtering yielded 32,177 DEGs (Fig. S5B; Table S4). Based on expression patterns, DEGs were categorized as HS-induced (up-regulated in > 70% of responsive genotypes), HS-repressed (down-regulated in > 70%), or “response-variable” (inconsistent regulation across genotypes). Of these, 12,817 (39.83%) were HS-induced, 15,872 (49.33%) were HS-repressed, and 3,488 (10.84%) response-variable (Fig. S5B). Strikingly, most DEGs exhibited consistent responses across the population: > 75% of both HS-induced and HS-repressed DEGs fell into Class C (differentially expressed in 90–100% of responsive accessions) (Fig. S5B), and their expression patterns are summarized in the heatmap (Fig. S5C).

Gene Ontology (GO) enrichment analysis revealed that HS-induced DEGs were predominantly associated with “response to heat”, “protein folding” and “response to hydrogen peroxide” (Fig. S5D). In contrast, HS-repressed DEGs were significantly enriched in biosynthetic and metabolic processes, such as “starch biosynthetic process” and “starch metabolic process” (Fig. S5E). These molecular signatures corroborate the observed reduction in TGW under HS and validate the physiological of our treatment.

**Fig. 3:**
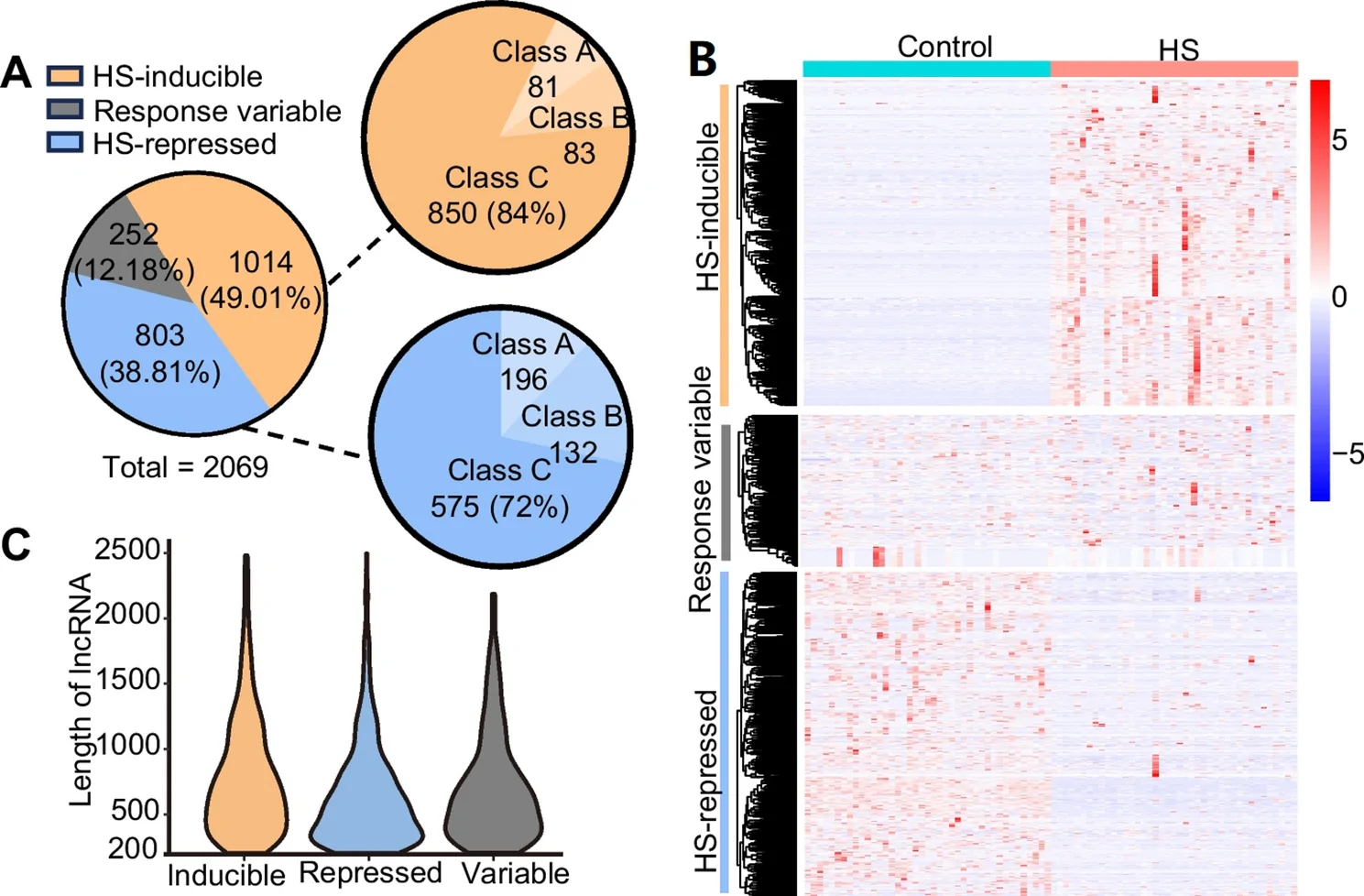
Expression dynamics and structural features of HS-responsive lncRNAs in wheat during grain filling. **A** Proportional classification of 2,069 differentially expressed lncRNAs (DELs) across response-variable, HS-induced, and HS-repressed clusters. Induced and repressed DELs are further stratified into three sensitivity classes: Class A (70% ≤ n < 80%), Class B (80% ≤ n < 90%), and Class C (90% ≤ n ≤ 100%), where n represents the percentage of genotypes displaying significant differential expression. **B** Expression profiles of 2,069 DELs across the 41 wheat genotypes in response to HS. Colors represent scaled, normalized expression levels (Z-scores of log₂-transformed FPKM values). **C** Length distribution of DELs within the three distinct HS-responsive expression clusters. Bar represent the absolute counts of lncRNAs within specified transcript length intervals

For lncRNAs, we applied independent criteria: a transcript was classified as a DEL if |log₂(fold change)|≥ 1 and it was differentially expressed in > 12.2% (5/41) of genotypes. This filtering identified 2,069 DELs (Fig. 3A; Table S5). Following the classification scheme used for DEGs, 1,014 (49.01%) were HS-induced, 803 (38.81%) were HS-repressed, and 252 (12.18%) were response-variable (Fig. 3A). Compared to mRNAs, DELs showed a lower proportion of HS-repressed transcripts but higher proportions of HS-induced and response-variable transcripts. A heatmap visualization illustrated their expression patterns across genotypes (Fig. 3B). Consistent with the DEGs, the vast majority of DELs displayed uniform responses: 84% of HS-induced and 72% of HS-repressed DELs fell into Class C (Fig. 3A), supporting their potential involvement during HS adaptation.

Finally, we observed that HS-repressed lncRNAs were significantly enriched for shorter transcripts (< 500 bp) compared to other response categories (Fig. 3C).

### Co-expression network analysis of HS-responsive lncRNA and mRNA

**Fig. 4:**
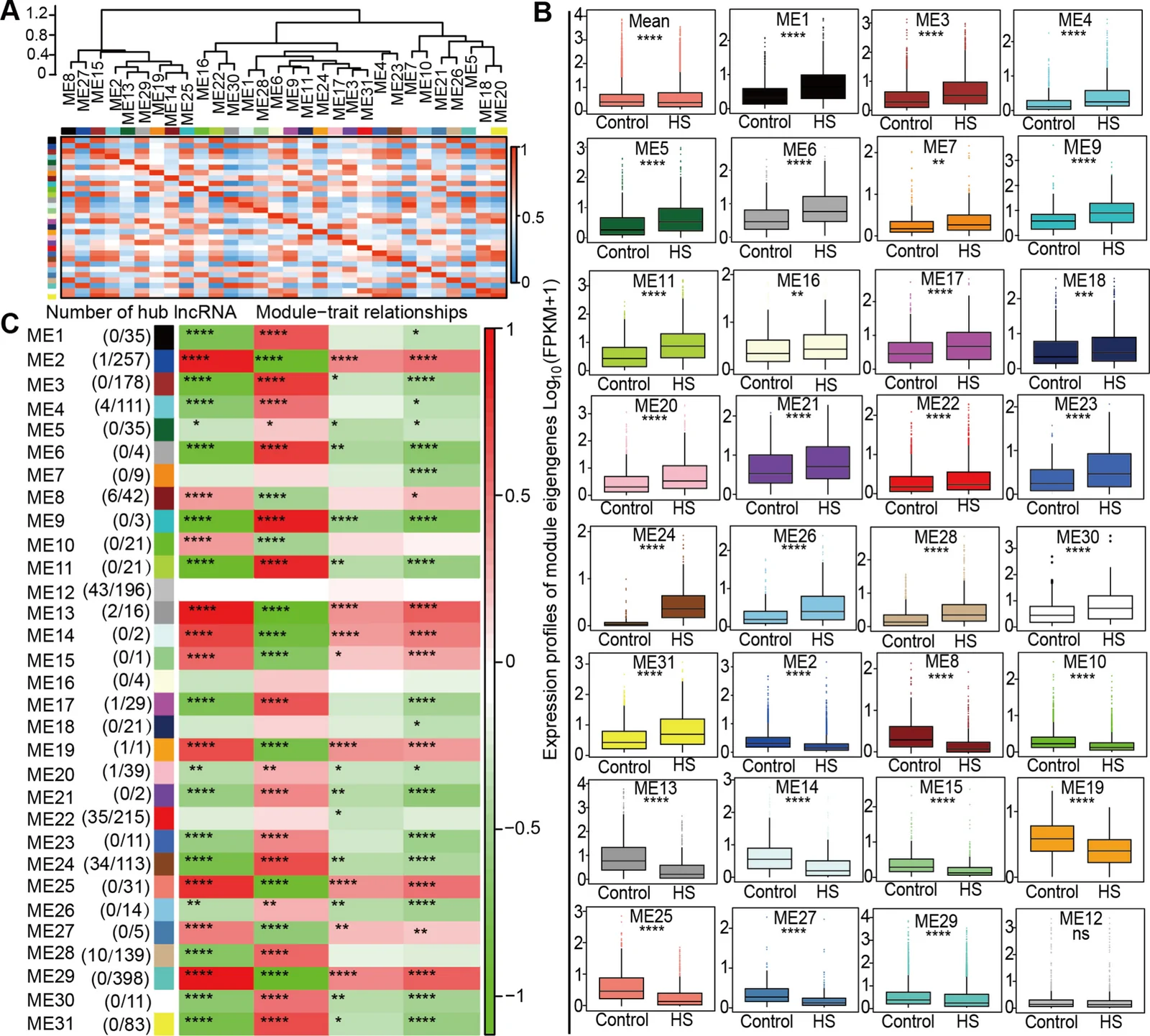
WGCNA identifying lncRNA-mRNA co-expressed modules significantly associated with HS. **A** Weighted gene co-expression network analysis (WGCNA) was used to identify discrete expression modules (ME1–ME31) containing both lncRNAs and mRNAs. The hierarchical clustering dendrogram shows the relationships among modules based on expression similarity. **B** Comparison of expression profiles for module eigengenes under control and HS conditions. Statistical significance was evaluated using a two-sided Wilcoxon rank-sum test (*P < 0.05; **P < 0.01; ***P < 0.001, ****P < 0.0001). **C** Evaluation of module association thousand-grain weight (TGW) phenotypes. The heatmap shows the Pearson correlation coefficients (*r*) between module eigengenes rows) and TGW values (columns) evaluated under control and HS conditions in field trials across the 2024 and 2025 cropping seasons. Color intensity and values indicate the correlation coefficient, with statistical significance thresholded at *P* < 0.05

To elucidate the potential coordinated regulatory networks involving lncRNAs and mRNAs during the HS response, we performed a weighted gene co‑expression network analysis (WGCNA) integrating all 2,029 DELs and 32,177 DEGs. The analysis grouped 2,045 lncRNAs and 32,058 mRNAs into 31 modules (ME1-ME31), with lncRNA counts per module ranging from 1 to 398, and mRNA counts from 138 to 7,189 (Fig. 4A). Eigengene expression profiling revealed that 20 modules (ME1, ME3–ME7, ME9, ME11, ME16–ME18, ME20-ME24, ME26, ME28, ME30, and ME31) were significantly upregulated under HS, whereas 10 modules (ME2, ME4, ME8, ME10, ME13, ME17, ME19, ME20, ME22, ME24, and ME28) were significantly downregulated (Fig. 4B), indicating systemic transcriptional reprogramming.

To evaluate the biological relevance of these modules, we correlated their eigengene expression levels with TGW measured under control and HS conditions in field trials across 2024 and 2025. The expression of 27 modules correlated significantly with TGW (*P* < 0.05) (Fig. 4C), suggesting that genes within these modules are closely linked to thermotolerance during grain filling. Subsequently, we identified 79 hub lncRNAs based on high module membership (kME > 0.9), spanning 10 modules (ME2, ME4, ME8, ME13, ME17, ME19, ME20, ME22, ME24, and ME28) (Table S6).The highest concentrations of hub lncRNAs were observed in modules ME22 (35 lncRNAs), ME24 (34), and ME28 (10) (Fig. 4C).

**Fig. 5:**
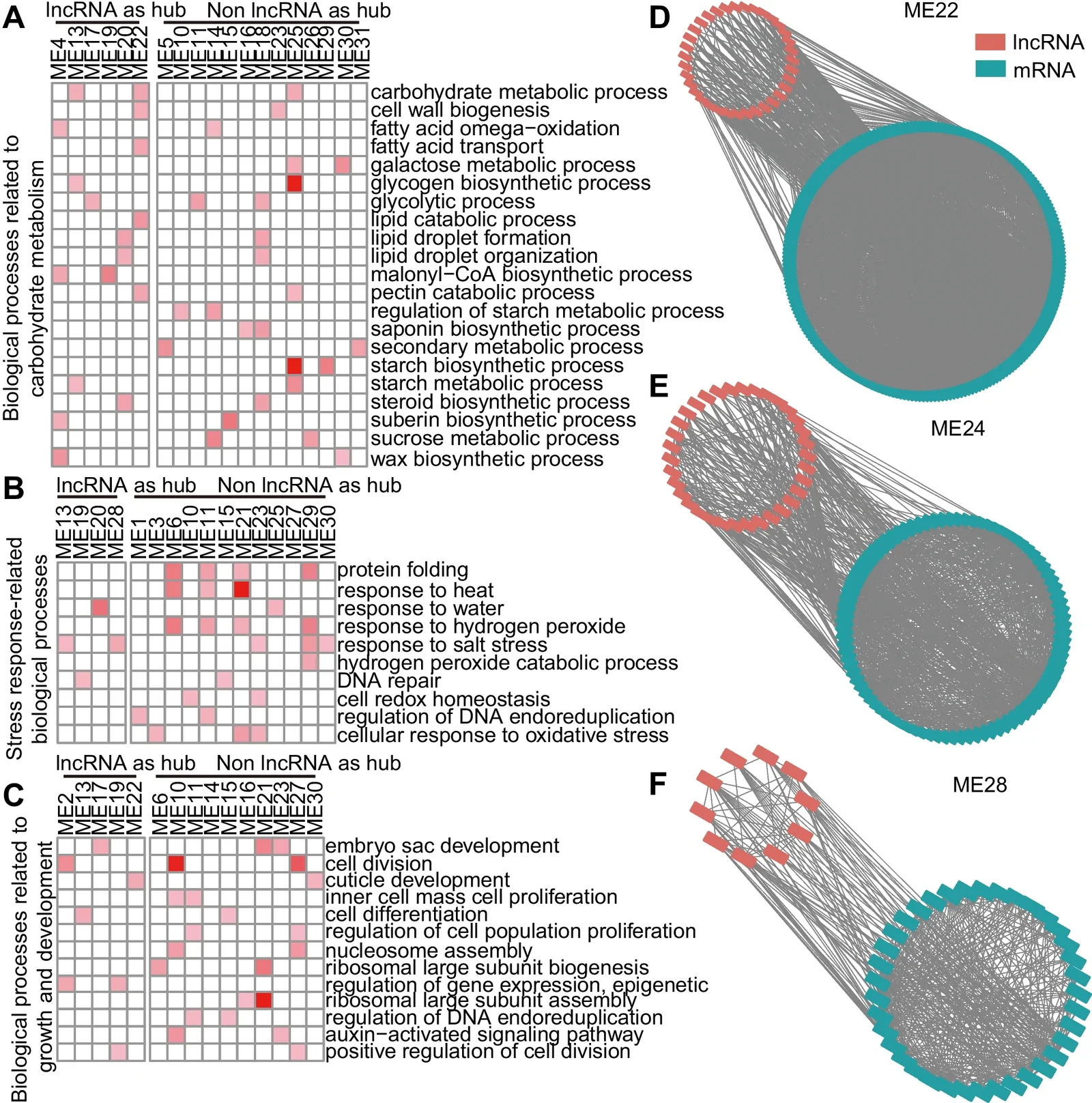
Functional Gene Ontology (GO) enrichment and co-expression interaction networks of prioritized modules. **A** GO enrichment analysis of genes within each module, showing significant enrichment of carbohydrate metabolism- and cuticle development-related terms. **B** GO enrichment analysis of genes in each module, showing significant enrichment of stress-responsive terms, including response to heat, water, hydrogen peroxide, salt stress, and cellular redox homeostasis. **C** GO enrichment analysis of genes in each module, highlighting terms related to reproductive development and cellular proliferation, such as embryo sac development, cell division, cuticle development, auxin signaling, and regulation of DNA endoreduplication. **D**–**F** Transcriptional co-expression networks outlining interactions between hub lncRNAs and hub mRNAs in the ME22 (**D**), ME24 (**E**), and ME28 (**F**) modules. Selected annotations of key biological pathways are highlighted for target network clusters

GO enrichment analysis indicated that genes in module ME22 were associated with “carbohydrate metabolic process” and “cuticle development” (Fig. 5A, C), while genes in module ME28 were enriched in salt‑stress‑related terms (Fig. 5B). These findings imply that these modules coordinately participate in environmental stress response, seed development, and growth regulation. Furthermore, network visualization illustrated close co-expression relationships between hub lncRNAs and mRNAs within modules ME22, ME24, and ME28 (Fig. 5D–F).

To assess the genetic relevance of 79 hub lncRNAs, We compared their genomic locations against the previously reported thermotolerance-associated meta-QTLs (MQTLs) [49]. Remarkably, 40 hub lncRNAs (50.6%) spatially overlapped with known MQTL regions (Table S7). This substantial overlap reinforces the biological relevance of these candidate loci in heat adaptation.

### Association of lncRNA haplotypes with TGW

To investigate potential genetic linkages between lncRNA natural variation and thermotolerance during grain filling, we analyzed genetic variations within the 79 hub lncRNA loci. Utilizing exome sequencing data and TGW phenotypes collected across eight environments from a wheat natural population [50], we inferred haplotype structures and evaluated their associations with TGW using mixed linear model (MLM). This model was corrected for both population structure (Q) and kinship algorithms to conservatively estimate associations.

**Fig. 6:**
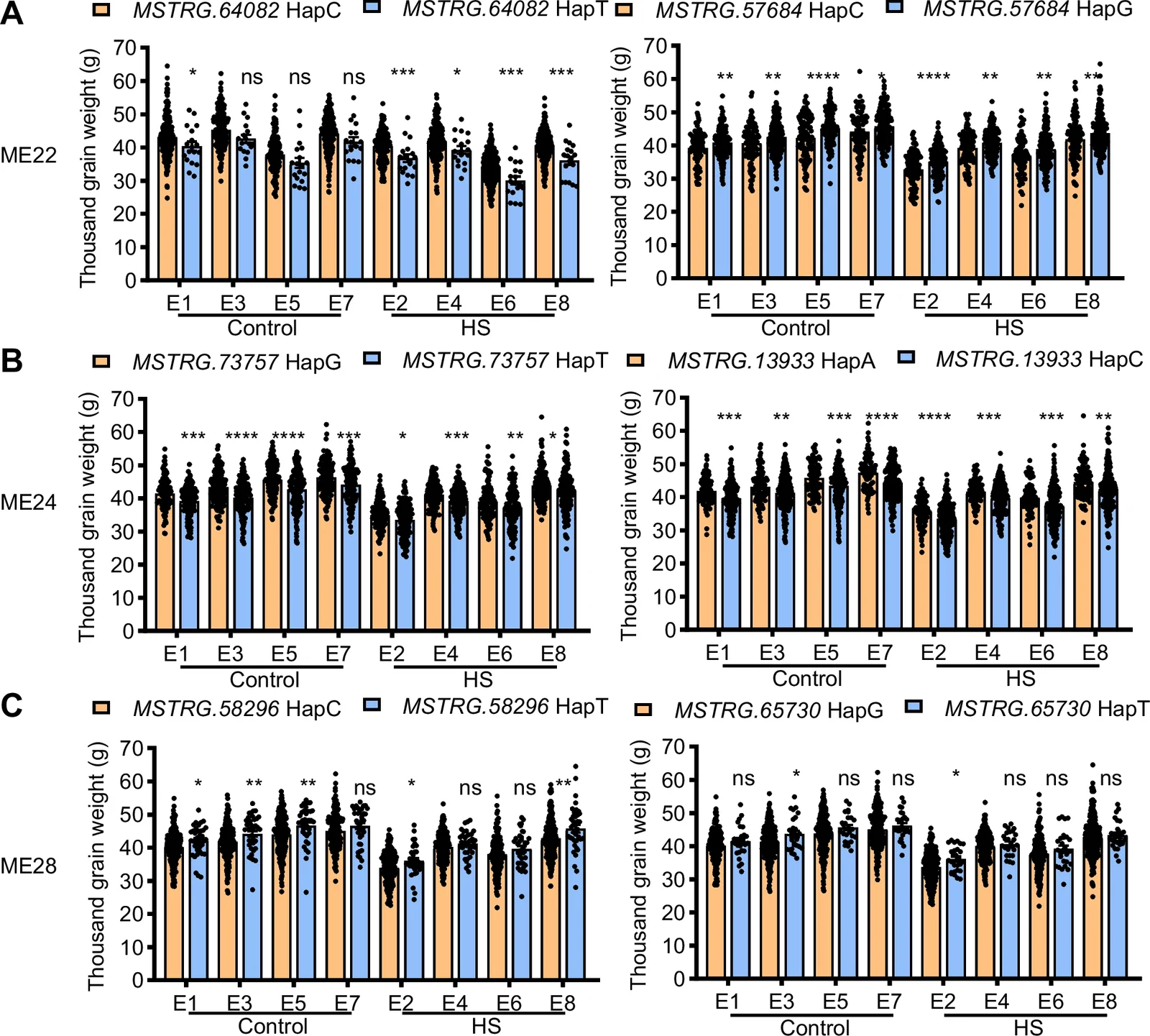
Genetic association of hub lncRNA haplotypes with TGW under eight multiple environmental conditions. **A**–**C** TGW comparison among different haplotypes for selected hub lncRNAs from modules ME22 (**A**: *MSTRG.64082* and *MSTRG.57684*), ME24 (**B**: *MSTRG.73757* and *MSTRG.13933*), and ME28 (**C**: *MSTRG.58296* and *MSTRG.65730*) under eight environmental conditions. Each point represents a single wheat accession from a natural population of 323 accessions. Data are presented as mean ± SEM. Statistical significance was determined using a two-sided Student’s *t*-test (*P < 0.05; **P < 0.01; ***P < 0.001, ****P < 0.0001, ns: not significant). E1 to E8 denote environmental conditions at Zhao County in 2021 under drought stress (D), drought and heat stress (DH), well-watered (W), well-watered and heat stress (WH) and 2022 under D, DH, W, and WH, respectively

Within module ME22, haplotypes of six hub lncRNAs showed significant associations with TGW. Notably, haplotypes of *MSTRG.64082* were significantly associated with TGW in all eight environments (Table 1). Specifically, HapC of *MSTRG.64082* correlated with significantly higher TGW under HS conditions, with no detectable negative effect on yield under control conditions. In contrast, *MSTRG.57684* HapG was associated with higher TGW under both normal and HS conditions (Fig. 6A). This suggests that *MSTRG.64082* HapC may serve as a favorable allele contributing specifically to thermotolerance. In module ME24, haplotypes of 11 hub lncRNAs were significantly associated with TGW, with *MSTRG.13933* and *MSTRG.73757* exhibiting consistent associations across all eight environments (Table 2). Both *MSTRG.73757* HapG and *MSTRG.13933* HapA were constitutively associated with higher TGW regardless of stress (Fig. 6B). Conversely, in module ME28, although five hub lncRNA haplotypes showed significant associations, their effects appeared environment-specific, being detected in only one to three environments (Table 3). For instance, *MSTRG.58296* HapT and *MSTRG.65730* HapT were linked to higher TGW only under specific environmental conditions (Fig. 6C).

**Table 1:** Association of hub lncRNA haplotypes in the ME22 module with TGW across eight environments in 323 wheat accessions

Environment		E1	E2	E3	E4	E5	E6	E7	E8
Years		2021	2021	2021	2021	2022	2022	2022	2022
Location		Zhao County	Zhao County	Zhao County	Zhao County	Zhao County	Zhao County	Zhao County	Zhao County
Water and temperature		D	DH	W	WH	D	DH	W	WH
*P*-value	MSTRG.64082	2.5594E-06	3.5454E-06	0.00727	0.01153	9.93E-03	1.71E-03	2.03E-02	0.01386
	MSTRG.79795	0.00507	0.00068151	0.01705	0.03167	0.00496	0.00449	0.00195	ns
	MSTRG.57684	ns	0.0029	0.02335	0.00215	0.00059361	ns	ns	0.04366
	MSTRG.62209	3.94E-01	0.03949	ns	ns	ns	ns	ns	ns
	MSTRG.103497	3.28E-01	ns	ns	ns	ns	0.01358	ns	ns
	MSTRG.104881	ns	ns	0.02446	4.44E-01	ns	ns	ns	ns

E1 to E8 indicate the environmental conditions at Zhao County across 2021 and 2022 representing combinations of water regimes (D: drought stress, W: well-watered) and temperature regimes (H: heat stress)

**Table 2:** Association of hub lncRNA haplotypes in the ME24 module with TGW across eight environments in 323 wheat accessions

Environment		E1	E2	E3	E4	E5	E6	E7	E8
Years		2021	2021	2021	2021	2022	2022	2022	2022
Location		Zhao County	Zhao County	Zhao County	Zhao County	Zhao County	Zhao County	Zhao County	Zhao County
Water and temperature		D	DH	W	WH	D	DH	W	WH
*P*-value	MSTRG.13933	7.9109E-06	1.8824E-06	0.00064801	0.000025815	2.09E-05	3.49E-05	1.70E-06	0.00062814
	MSTRG.73757	5.7287E-06	0.00214	3.4662E-09	0.0001026	5.4715E-08	0.00039941	0.000065418	0.00452
	MSTRG.26767	0.00396	ns	0.01072	0.00078301	0.00013821	0.03632	0.00649	0.01651
	MSTRG.65220	4.45E-03	0.04563	0.02905	ns	0.00324	0.00958	ns	0.03054
	MSTRG.111121	2.20E-03	0.01919	0.00112	0.00021438	0.04326	ns	ns	ns
	MSTRG.79113	ns	0.04527	0.23611	4.93E-01	0.03214	0.03745	ns	ns
	MSTRG.65213	0.03334	0.01267	0.02771	ns	ns	ns	ns	ns
	MSTRG.33350	0.02532	0.00468	ns	ns	ns	ns	ns	ns
	MSTRG.115134	ns	0.01081	ns	ns	ns	0.00445	ns	ns
	MSTRG.2503	ns	0.04375	ns	ns	ns	ns	ns	ns
	MSTRG.31728	ns	ns	ns	ns	ns	0.009	ns	ns

E1 to E8 indicate the environmental conditions at Zhao County across 2021 and 2022 representing combinations of water regimes (D: drought stress, W: well-watered) and temperature regimes (H: heat stress)

**Table 3:** Association of hub lncRNA haplotypes in the ME28 module with TGW across eight environments in 323 wheat accessions

Environment		E1	E2	E3	E4	E5	E6	E7	E8
Years		2021	2021	2021	2021	2022	2022	2022	2022
Location		Zhao County	Zhao County	Zhao County	Zhao County	Zhao County	Zhao County	Zhao County	Zhao County
Water and temperature		D	DH	W	WH	D	DH	W	WH
*P*-value	MSTRG.58296	ns	ns	0.02542	ns	0.03201	ns	ns	0.01289
	MSTRG.65730	ns	0.0209	0.04043	ns	ns	ns	ns	ns
	MSTRG.126033	0.00915	ns	ns	ns	0.00768	ns	ns	ns
	MSTRG.87618	ns	ns	0.03451	ns	ns	ns	ns	ns
	MSTRG.123175	ns	ns	0.03098	ns	ns	ns	ns	ns

E1 to E8 indicate the environmental conditions at Zhao County across 2021 and 2022 representing combinations of water regimes (D: drought stress, W: well-watered) and temperature regimes (H: heat stress)

In summary, while variations in *MSTRG.57684*, *MSTRG.73757*, and *MSTRG.13933* consistently correlated with higher TGW across diverse environments, variation in *MSTRG.64082* was preferentially associated with TGW maintenance under HS. Therefore, *MSTRG.64082* HapC emerges as a putative favorable haplotype for stabilizing TGW under HS.

### Expression and phenotypic association of MSTGR.64082 in thermotolerance

**Fig. 7:**
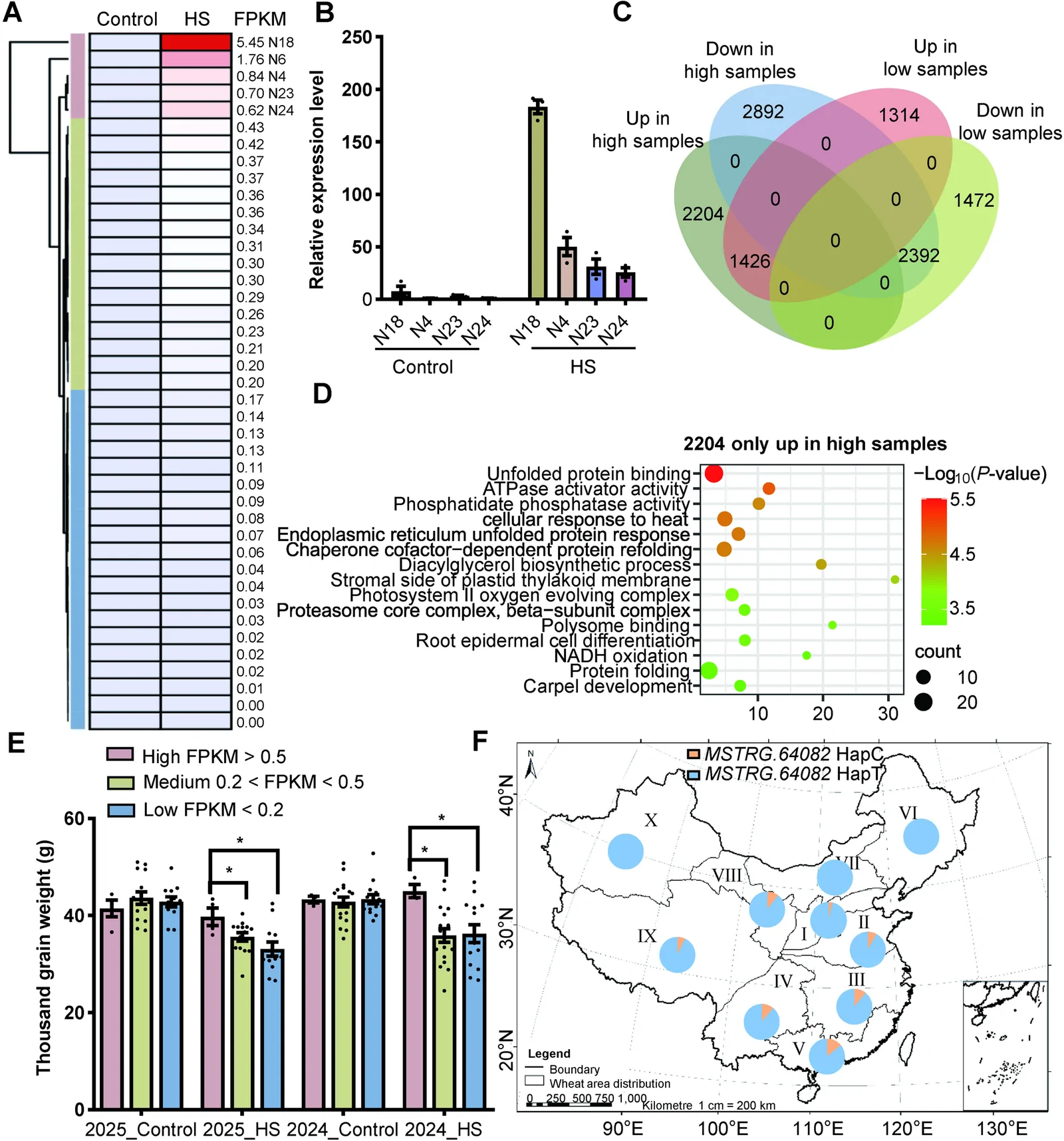
Expression and phenotypic association analysis of the candidate lncRNA *MSTRG.64082*. **A** Heatmap of MSTRG.64082 expression levels (FPKM) across 41 wheat genotypes under normal and HS conditions, categorized by hierarchical clustering into High-, Medium-, and Low-expression groups. **B** RT-qPCR validation of *MSTRG.64082* expression during grain filling in grains of for representative genotypes (N18, N4, N23, and N24) under control and HS conditions. Relative expression normalized to the internal reference gene *TaActin*. **C** Venn diagrams showing the extent of overlap among HS-responsive protein-coding genes in High-expression and Low-expression groups in response to HS. **D** GO term enrichment analysis of 2,204 genes exclusively upregulated in the High expression group. Circle size indicates gene count; color represents statistical significance (− log₁₀[*P*-value]). **E** Comparison TGW among the High-, Medium-, and Low-expression groups under control and HS conditions in field trials at Hengshui in 2024 and 2025. Each point represents a wheat genotype; data are shown as mean ± SEM.* P*-values were calculated using a two-sided Student’s *t*-test (*P < 0.05). **F** Geographical distribution of *MSTRG.64082* haplotypes in 217 Chinese landraces. I–X represent major wheat ecological cultivation zones: I, Northern Winter Wheat Zone; II, Yellow and Huai River Valleys Facultative Wheat Zone; III, Middle and Lower Yangtze Valleys Autumn-Sown Spring Wheat Zone; IV, Southwestern Autumn-Sown Spring Wheat Zone; V, Southern Autumn-Sown Spring Wheat Zone; VI, Northeastern Spring Wheat Zone; VII, Northern Spring Wheat Zone; VIII, Northwestern Spring Wheat Zone; IX, Qinghai-Tibetan Plateau Spring-Winter Wheat Zone; X, Xinjiang Winter-Spring Wheat Zone

To further explore the relationship between *MSTGR.64082* and thermotolerance during grain filling, we profiled its expression across the 41 wheat genotypes. The transcript was virtually undetectable under control conditions but was variably induced by HS (Fig. 7A). Based on expression levels (FPKM), the 41 wheat genotypes were classified into three groups: High (FPKM > 0.5, 5 genotypes), Medium (0.2 < FPKM < 0.5), and Low (FPKM < 0.2) (Fig. 7A). RT-qPCR analysis in four representative genotypes (N18, N4, N23, and N24) corroborated the RNA-seq data, confirming that *MSTRG.64082* is significantly induced by HS, with the strongest induction observed in N18 (Fig. 7B).

Comparative transcriptomics between High- and Low-expression groups revealed 2,204 genes that were uniquely upregulated in the High group (Fig. 7C). These genes were significantly enriched in GO terms such as "cellular response to heat" and "unfolded protein binding" (Fig. 7D), suggesting an association between *MSTRG.64082* activation and robust HS signaling. Field phenotyping demonstrated that under HS, the High-expression group exhibited significantly higher TGW than both the Medium and Low groups A positive correlation between TGW and the expression level of *MSTRG.64082* was also clearly evident in the 2025 field data (Fig. 7E). In contrast, no significant differences in TGW were observed among the three groups under normal conditions (Fig. 7E), indicating that elevated *MSTRG.64082* expression is specifically linked yield protection under HS.

Finally, we assessed the geographical distribution of *MSTRG.64082* haplotypes across 217 Chinese wheat landraces representing major wheat-growing regions [51, 52]. We observed a discernible latitudinal cline: HapT predominated in cooler, high-latitude regions, whereas the frequency of HapC increased significantly at lower latitudes corresponding to warmer climates (Fig. 7F). This clinal distribution aligns with potential environmental adaptation, supporting the hypothesis that *MSTRG.64082* HapC has been subjected to selection pressure in heat-prone regions.

## Discussion

Although long non-coding RNAs (lncRNAs) have garnered increasing attention regarding plant stress response, research focusing on the grain-filling stage—a critical determinant of final yield—remains scarce. The existing literature has predominantly concentrated on heat stress (HS) responses during pollen development or the seedling stage [53], leaving our understanding of lncRNA-associated regulatory networks during grain filling largely unexplored. To address this gap, we leveraged population-scale transcriptomics from 41 wheat genotypes and identified 2,069 HS-responsive lncRNAs. Intriguingly, only 12.18% of these lncRNAs exhibited variable response patterns among accessions; the vast majority displayed highly conserved expression dynamics. This expression paradigm closely mirrors that of mRNAs, suggesting that lncRNAs are integrated components of the core transcriptional reprogramming regulating the HS response at this developmental juncture.

In previous studies, the screening of functional lncRNAs has relied heavily on differential expression analysis alone. In our study, we adopted an integrative strategy combining weighted gene co-expression network analysis (WGCNA) with candidate gene-based genotype–phenotype association analysis. This methodology expands upon traditional expression-based screens by simultaneously contextualizing transcriptional co-regulation networks and natural genomic variations, thereby facilitating the systematic prioritization of key candidate loci. As validation, we found that over half (50.6%) of our 79 identified hub lncRNAs spatially matched previously reported thermotolerance-associated MQTLs regions [49]. This convergence of transcriptomic and historical QTL data robustly supports the biological relevance of these prioritized loci.

Through this dual-evidence approach, we identified *MSTRG.64082* as a prominent candidate. Its expression exhibited a strong positive correlation with thousand-grain weight (TGW) (Fig. 7C), and specific genetic variants within its locus were statistically associated with heat tolerance traits (Fig. 6A). Comparative analysis indicated that accessions with high *MSTRG.64082* expression uniquely upregulated a suite of 2,204 genes significantly enriched for "cellular response to heat" (Fig. 7D). Furthermore, the population-level haplotype geographical distribution of *MSTRG.64082* displayed a clear latitudinal pattern: with the beneficial HapC allele being significantly enriched in warmer, lower-latitude climates (Fig. 7F). Together, these transcriptomic, genetic, and ecological data strongly imply that *MSTRG.64082* acts as a positive factor in wheat environmental adaptation, making it a valuable functional marker for breeding heat-resilient varieties.

Regarding potential molecular mechanisms, LncRNAs frequently as miRNA target mimics, acting as competing endogenous RNAs (ceRNAs) [21–24]. Preliminary in silico analysis using psRNATarget [54]predicted that *MSTRG.64082* may act as a target mimic for specific miRNA, hinting at an ncRNA–miRNA–mRNA regulatory module. However, given that lncRNAs employ highly diverse regulatory modalities—ranging from recruiting chromatin modifiers and interacting with transcription factors to mediating epigenetic and post-transcriptional control [16–18]—it is highly plausible that *MSTRG.64082* utilizes additional or alternative mechanisms. While our current findings establish a robust genetic and transcriptional association with thermotolerance, extensive biological experiments, such as CRISPR/Cas9-mediated knockout or transgenic overexpression, are fundamentally required in future studies to ascertain the precise mechanistic pathways governed by *MSTRG.64082*.

In summary, this study constructs a comprehensive co-expression network of HS-responsive lncRNAs during the wheat grain-filling stage and successfully highlights a promising candidate locus, *MSTRG.64082*. By integrating sequence variation data with pan-transcriptomic expression profiling, we provide compelling correlative evidence for the role of *MSTRG.64082* in wheat heat adaptation. This resource not only broadens our understanding of the non-coding transcriptome in crop stress biology but also delivers actionable genetic targets for the molecular breeding of climate-resilient wheat.

## Methods

### Plant materials and data collection

A collection of 41 representative wheat genotypes (Table S1) were cultivated at the experimental station Hengshui (37°44′ N, 115°40′ E) China, during the 2024–2025 cropping season. Crucially, all genotypes were grown under identical soil conditions and microclimatic environments within the same field trial. To simulate heat stress (HS) during the grain-filling stage, a controlled field experiment was conducted: polythene covers were installed over the designated heat-treatment plots approximately 14 days post-anthesis (DPA) to elevate the temperature. For phenotypic evaluation, thousand-grain weight (TGW) was measured under both control and HS conditions using five randomly selected individuals per genotype at physiological maturity.

### RNA sequencing and data processing

On the 3rd day of the HS treatment (corresponding to 17 DPA), developing grains from the middle section of three spikes, each harvested from physically separate individuals per genotype, were pooled to construct a representative biological sample for subsequent RNA sequencing. This pooling strategy was employed to minimize intra-genotypic microenvironmental variation. All field samples were collected from 9:00 AM to 11:00 AM to minimize circadian effects. Total RNA of was extracted using the Plant Tissue RNA Isolation kit (Qiagen, Germany) according to the manufacturer’s instructions. High-throughput RNA sequencing (RNA-seq) was conducted on the Illumina® HiSeq NextSeq 2000 platform (BioMarker, China) using a 150-bp paired-end sequencing strategy. A total of 82 RNA-seq libraries (41 genotypes × 2 conditions) were sequenced, yielding approximately 80 Gb of clean sequence data.

Raw sequencing reads were evaluated using FastQC v0.11.9 (https://www.bioinformatics.babraham.ac.uk/projects/fastqc/). Low-quality reads, adapter sequences, and poly-N-containing reads were trimmed using fastp v0.20.1 (https://anaconda.org/bioconda/fastp) with default parameters. High-quality clean reads were mapped to the reference genome of wheat cultivar KN9204 [55] using HISAT2 v2.1.0 [56] with default parameters, permitting up to one mismatch for 75-bp reads and three mismatches for 150-bp reads. Only uniquely mapped reads were retained and sorted using SAMtools v1.9 [57] for downstream transcript assembly.

### Identification of lncRNAs

Following alignment, transcript assembly was performed using StringTie v2.1.4 [58] to construct both reference-guided and de novo transcripts with default settings. Assembled transcripts were compared against the reference annotation (KN9204) using Gffcompare [59] to classify transcript isoforms. Only novel transcripts matching Gffcompare class codes “i” (completely containing a reference intron) and “u” (intergenic transcripts with no reference gene overlap) were selected for further lncRNA screening. Candidate transcripts shorter than 200 nucleotides were excluded. To verify their non-coding nature, the retaining transcripts were evaluated using three independent coding-potential prediction tools run with default parameters: CPC2 v1.0.1 [60] (predicting “non-coding”), CNCI v2.0 [61] (index score < 0), and PLEK [62] ("non-coding" label). Transcripts that were consistently flagged as non-coding by all three analytical pipelines were defined as high-confidence lncRNAs.

### Differential expression and pan-transcriptome analysis

Normalized expression levels (Fragments Per Kilobase of transcript per Million mapped reads, FPKM) for both mRNAs and lncRNAs were estimated using StringTie v2.1.4 [58] with the flags -e -G. To identify differentially expressed genes (DEGs) and lncRNAs (DELs) responsive to HS, pairwise differential expression analysis was performed separately for each of the 41 genotypes using the R package edgeR (https://bioconductor.org/packages//2.11/bioc/html/edgeR.html). To address the lack of within-cultivar biological sequencing replicates, we estimated biological variation by assigning a conservative, fixed biological coefficient of variation (BCV) value of 0.2, as recommended by the edgeR user manual for unreplicated designs. Transcripts exhibiting an absolute log₂‑fold change (|log₂FC|) ≥ 1 and a false discovery rate (FDR)‑adjusted *P*‑value < 0.05 were defined as significantly differentially expressed.

### Co-expression modules construction

To identify co-expression networks with the HS response weighted gene co-expression network analysis (WGCNA) was performed using the WGCNA package in R [63]. A total 2,029 DELs and 32,177 DEGs were utilized as the input dataset. A soft-thresholding power of *β* = 9 was selected based on the scale‑free topology criterion (model fit index R^2^ > 0.90). The resulting adjacency matrix was converted into a signed Topological Overlap Matrix (TOM) to measure network interconnectedness. Modules were detected using the blockwiseModules function with the following parameter settings: power = 9, TOMType = “signed”, minModuleSize = 30, mergeCutHeight = 0.25. Modules exhibiting highly correlated eigengenes (*r* > 0.75) were merged. Unassigned transcripts were grouped into the ‘grey’ module and excluded from downstream analysis. Module eigengenes (MEs), representing the first principal component of module expression profiles, were calculated to correlate network expression with TGW phenotypes. Hub lncRNAs were identified based on a high module membership threshold (kME) > 0.9. Finally, node and edge attribute files representing core interactions within selected modules were exported for network visualization using Cytoscape [64].

### Haplotype and TGW phenotypic association analysis

Haplotype variants for the 79 identified hub lncRNAs were extracted from a publicly available exome sequencing dataset consisting of 323 wheat accessions [50]. The associated phenotypic dataset comprised TGW values evaluated across eight independent environments (comprising four water‑heat regimes over the 2021–2022 cropping season). Haplotype-phenotype association analysis was conducted using TASSEL v5.0. To minimize the risk of false-positive associations, we implemented a mixed linear model (MLM) that accounted for both population structure (*Q*-matrix derived from the first three principal components) and genetic relatedness (*K*-matrix, Kinship). Only haplotype associations that remained statistically significant (*P* < 0.05) after multi-factorial MLM correction were reported as stable genetic associations.

### RT‐qPCR and gene expression analysis

Total RNA was extracted from harvested developing grains (collected during the grain-filling stage) using TRIzol reagent (Invitrogen), adhering strictly to the manufacturer’s protocol. First‐strand complementary DNA (cDNA) was synthesized from 1μg of total RNA using the HiScript II Q RT SuperMix for qPCR (+ gDNA wiper) kit (Vazyme; R223-01) to eliminate genomic DNA contamination. Real-time quantitative PCR (RT‐qPCR) was performed with the ChamQ Universal SYBR qPCR Master Mix (Vazyme; Q711-02) on a CFX384 Touch Real-Time PCR Detection System (Bio‐Rad, USA). Specific primers for LncRNA *MSTRG.64082* (Forward: 5’-ATGTCCCTGCAAGCCATCACCG-3’; Reverse: 5’-CTGCCGGTCAGATACACCGTCAATACG-3’) were designed. The wheat *TaActin*, gene (primers: Forward: 5’-CTCCCTCACAACAACAACCGC-3’; Reverse: 5’-TACCAGGAACTTCCATACCAAC-3’) was utilized as an internal housekeeping reference. Thermal cycling conditions and the melting curve profile were set according to the manufacturer's default instructions. Relative transcript expression levels were calculated using the comparative threshold cycle (2^−ΔΔCq^) method [65].

## Supplementary Information

Supplementary Material 1: Figure S1. Heat stress significantly reduces grain weight during wheat grain filling. Dynamic profiles of grain dry weight (g per 200 grains) of the elite wheat cultivar KN9204 during grain‑filling stage under control and HS conditions. Data represent mean ± SEM from at least three independent biological replicates.

Supplementary Material 2: Figure S2. Population structure of the 41 wheat genotypes. A: Estimated stacking diagram structure, B: PCA plot, and C: phylogenetic trees showing the population structure of the 41 wheat genotypes used in this study. The stacking diagram structure of the population with K = 2-5. A vertical line represents each accession, and population types indicated by different colors. The PCA plot of the wheat accessions were based on two principal components accounts for most overall variability. The phylogenetic trees of the 41 wheat genotypes constructed using the Neighbor-Joining method. Tree scale = 0.1. 

Supplementary Material 3: Figure S3. Bioinformatic analysis workflow for systemic lncRNA identification. Overview of the bioinformatic workflow used for systematic lncRNA identification. RNA-seq data generated from developing grains (21 days after flowering, DAF) of 41 wheat genotypes under control and HS conditions. The pipeline comprised transcriptome assembly, followed by the removal of protein‑coding transcripts, known annotations, and short transcripts (length < 200 nt).

Supplementary Material 4: Figure S4. Comparative characterization of lncRNAs and mRNAs structural properties. A: Density distribution of exon numbers per transcript in lncRNAs and mRNAs. B: Length distribution of lncRNAs and mRNAs (bp). C: Global expression levels [log₁₀(FPKM+1)] of lncRNAs and mRNAs under control and HS conditions. D: Number of samples (wheat genotypes) expressing lncRNAs and mRNAs under control and HS conditions.

Supplementary Material 5: Figure S5. Quantitative distribution and functional categorization of HS-responsive genes in wheat. A: Number of wheat cultivars exhibiting differentially expressed lncRNAs or mRNAs in response to HS. B: Percentages and absolute counts of 32,177 DEGs partitioned into response-variable, HS-induced, and HS-repressed groups, further stratified into Class A, B, and C, where n represents the percentage of genotypes showing significant differential expression. C: Heatmap showing the expression patterns of 32,177 DEGs across genotypes under HS. Color intensity corresponds to normalized expression level (log‑scale). D, E: Enriched Gene Ontology (GO) terms for HS‑induced mRNA (D) and HS‑repressed mRNAs (E).

Supplementary Material 6.

## Data Availability

The RNA sequencing data generated in this study have been deposited in the Genome Sequence Archive (GSA) at the National Genomics Data Center, China National Center for Bioinformation/Beijing Institute of Genomics, Chinese Academy of Sciences, under accession number CRA038950. The data are accessible via (https://ngdc.cncb.ac.cn/gsa/s/5q552AaK).

## References

[1] Su P, Jiang C, Qin H, Hu R, Feng J, Chang J, et al. Identification of potential genes responsible for thermotolerance in wheat under high temperature stress. Genes. 2019;10:174. 10.3390/genes10020174. PMC6410297 30823586

[2] Shirdelmoghanloo H, Chen K, Paynter BH, Angessa TT, Westcott S, Khan HA, et al. Grain-filling rate improves physical grain quality in barley under heat stress conditions during the grain-filling period. Front Plant Sci. 2022;13:858652. 10.3389/fpls.2022.858652. PMC9137397 35645996

[3] Wang X, Hou L, Lu Y, Wu B, Gong X, Liu M, et al. Metabolic adaptation of wheat grain contributes to a stable filling rate under heat stress. J Exp Bot. 2018;69:5531–45. 10.1093/jxb/ery303. PMC6255704 30476278

[4] Wang H, Feng M, Jiang Y, Du D, Dong C, Zhang Z, et al. Thermosensitive SUMOylation of TaHsfA1 defines a dynamic ON/OFF molecular switch for the heat stress response in wheat. Plant Cell. 2023;35:3889–910. 10.1093/plcell/koad192. PMC10533334 37399070

[5] Hu X-J, Chen D, Lynne Mclntyre C, Fernanda Dreccer M, Zhang Z-B, Drenth J, et al. Heat shock factor C2a serves as a proactive mechanism for heat protection in developing grains in wheat via an ABA-mediated regulatory pathway. Plant Cell Environ. 2018;41:79–98. 10.1111/pce.12957. 28370204

[6] Wei J-T, Zheng L, Ma X-J, Yu T-F, Gao X, Hou Z-H, et al. An ABF5b-HsfA2h/HsfC2a-NCED2b/POD4/HSP26 module integrates multiple signaling pathway to modulate heat stress tolerance in wheat. Plant Biotechnol J. 2025;23:4735–51. 10.1111/pbi.70164. PMC12576446 40693724

[7] Zhang R, Liu G, Xu H, Lou H, Zhai S, Chen A, et al. Heat stress tolerance 2 confers basal heat stress tolerance in allohexaploid wheat (*Triticum aestivum* L.). J Exp Bot. 2022;73:6600–14. 10.1093/jxb/erac297. 35781562

[8] Wilhelm EP, Mullen RE, Keeling PL, Singletary GW. Heat stress during grain filling in maize: effects on kernel growth and metabolism. Crop Sci. 1999;39:1733–41. 10.2135/cropsci1999.3961733x.

[9] Sumesh KV, Sharma-Natu P, Ghildiyal MC. Starch synthase activity and heat shock protein in relation to thermal tolerance of developing wheat grains. Biol Plant. 2008;52:749–53. 10.1007/s10535-008-0145-x.

[10] Thitisaksakul M, Jiménez RC, Arias MC, Beckles DM. Effects of environmental factors on cereal starch biosynthesis and composition. J Cereal Sci. 2012;56:67–80. 10.1016/j.jcs.2012.04.002.

[11] Hurkman WJ, McCue KF, Altenbach SB, Korn A, Tanaka CK, Kothari KM, et al. Effect of temperature on expression of genes encoding enzymes for starch biosynthesis in developing wheat endosperm. Plant Sci. 2003;164:873–81. 10.1016/S0168-9452(03)00076-1.

[12] undefined. Shifting the limits in wheat research and breeding using a fully annotated reference genome. Science. 2018;361:eaar7191. 10.1126/science.aar7191. 30115783

[13] Zhang YC, He RQ, Cheng Y, Wang D, Ariel F, Chen YQ. Long noncoding RNAs as molecular architects: shaping plant functions and physiological plasticity. Mol Plant. 2025;18:1643–71. 10.1016/j.molp.2025.09.008. 40931525

[14] Baev V, Gisel A, Minkov I. The fascinating world of plant non-coding RNAs. Int J Mol Sci. 2023;24:10341. 10.3390/ijms241210341. PMC10299162 37373488

[15] Mattick JS, Amaral PP, Carninci P, Carpenter S, Chang HY, Chen L-L, et al. Long non-coding RNAs: definitions, functions, challenges and recommendations. Nat Rev Mol Cell Biol. 2023;24:430–47. 10.1038/s41580-022-00566-8. PMC10213152 36596869

[16] Chen W, Zhu T, Shi Y, Chen Y, Li WJ, Chan RJ, et al. An antisense intragenic lncRNA SEAIRa mediates transcriptional and epigenetic repression of SERRATE in Arabidopsis. Proc Natl Acad Sci. 2023;120:e2216062120. 10.1073/pnas.2216062120. PMC10013867 36857348

[17] Zhao X, Li J, Lian B, Gu H, Li Y, Qi Y. Global identification of Arabidopsis lncRNAs reveals the regulation of MAF4 by a natural antisense RNA. Nat Commun. 2018;9:5056. 10.1038/s41467-018-07500-7. PMC6265284 30498193

[18] Kindgren P, Ard R, Ivanov M, Marquardt S. Transcriptional read-through of the long non-coding RNA SVALKA governs plant cold acclimation. Nat Commun. 2018;9:4561. 10.1038/s41467-018-07010-6. PMC6212407 30385760

[19] Jin Y, Ivanov M, Dittrich ACN, Nelson ADL, Marquardt S. LncRNA FLAIL affects alternative splicing and represses flowering in *Arabidopsis*. EMBO J. 2023;42:EMBJ2022110921. 10.15252/embj.2022110921. PMC10233378 37051749

[20] Budak H, Kaya SB, Cagirici HB. Long non-coding RNA in plants in the era of reference sequences. Front Plant Sci. 2020;11:276. 10.3389/fpls.2020.00276. PMC7080850 32226437

[21] Cagirici HB, Alptekin B, Budak H. RNA sequencing and co-expressed long non-coding RNA in modern and wild wheats. Sci Rep. 2017;7:10670. 10.1038/s41598-017-11170-8. PMC5587677 28878329

[22] Budak H, Khan Z, Kantar M. History and current status of wheat miRNAs using next-generation sequencing and their roles in development and stress. Brief Funct Genomics. 2014;14:189–98. 10.1093/bfgp/elu021. 24962995

[23] Hussain B, Akpınar BA, Alaux M, Algharib AM, Sehgal D, Ali Z, et al. Capturing wheat phenotypes at the genome level. Front Plant Sci. 2022;13:851079. 10.3389/fpls.2022.851079. PMC9289626 35860541

[24] Zhang X, Shen J, Xu Q, Dong J, Song L, Wang W, et al. Long noncoding RNA lncRNA354 functions as a competing endogenous RNA of miR160b to regulate ARF genes in response to salt stress in upland cotton. Plant Cell Environ. 2021;44:3302–21. 10.1111/pce.14133. 34164822

[25] Ariel F, Jegu T, Latrasse D, Romero-Barrios N, Christ A, Benhamed M, et al. Noncoding transcription by alternative RNA polymerases dynamically regulates an auxin-driven chromatin loop. Mol Cell. 2014;55:383–96. 10.1016/j.molcel.2014.06.011. 25018019

[26] Henriques R, Wang H, Liu J, Boix M, Huang L-F, Chua N-H. The antiphasic regulatory module comprising CDF5 and its antisense RNA FLORE links the circadian clock to photoperiodic flowering. New Phytol. 2017;216:854–67. 10.1111/nph.14703. 28758689

[27] Sun Z, Huang K, Han Z, Wang P, Fang Y. Genome-wide identification of *Arabidopsis* long noncoding RNAs in response to the blue light. Sci Rep. 2020;10:6229. 10.1038/s41598-020-63187-1. PMC7148362 32277122

[28] Kim DH, Xi Y, Sung S. Modular function of long noncoding RNA, COLDAIR, in the vernalization response. PLoS Genet. 2017;13:e1006939. 10.1371/journal.pgen.1006939. PMC5552341 28759577

[29] Xu S, Dong Q, Deng M, Lin D, Xiao J, Cheng P, et al. The vernalization-induced long non-coding RNA VAS functions with the transcription factor TaRF2b to promote TaVRN1 expression for flowering in hexaploid wheat. Mol Plant. 2021;14:1525–38. 10.1016/j.molp.2021.05.026. 34052392

[30] Liu X, Li D, Zhang D, Yin D, Zhao Y, Ji C, et al. A novel antisense long noncoding RNA, TWISTED LEAF, maintains leaf blade flattening by regulating its associated sense R2R3-MYB gene in rice. New Phytol. 2018;218:774–88. 10.1111/nph.15023. 29411384

[31] Wang Y, Luo X, Sun F, Hu J, Zha X, Su W, et al. Overexpressing lncRNA LAIR increases grain yield and regulates neighbouring gene cluster expression in rice. Nat Commun. 2018;9:3516. 10.1038/s41467-018-05829-7. PMC6115402 30158538

[32] Fang J, Zhang F, Wang H, Wang W, Zhao F, Li Z, et al. Ef-cd locus shortens rice maturity duration without yield penalty. Proc Natl Acad Sci U S A. 2019;116:18717–22. 10.1073/pnas.1815030116. PMC6744900 31451662

[33] Zhou YF, Zhang YC, Sun YM, Yu Y, Lei MQ, Yang YW, et al. The parent-of-origin lncRNA MISSEN regulates rice endosperm development. Nat Commun. 2021;12:6525. 10.1038/s41467-021-26795-7. PMC8585977 34764271

[34] Ding J, Lu Q, Ouyang Y, Mao H, Zhang P, Yao J, et al. A long noncoding RNA regulates photoperiod-sensitive male sterility, an essential component of hybrid rice. Proc Natl Acad Sci U S A. 2012;109:2654–9. 10.1073/pnas.1121374109. PMC3289353 22308482

[35] Yang L, Cheng Y, Yuan C, Zhou YF, Huang QJ, Zhao WL, et al. The long non-coding RNA VIVIpary promotes seed dormancy release and pre-harvest sprouting through chromatin remodeling in rice. Mol Plant. 2025;18:978–94. 10.1016/j.molp.2025.04.010. 40275584

[36] Lei MQ, He RR, Zhou YF, Yang L, Zhang ZF, Yuan C, et al. The long noncoding RNA ALEX1 confers a functional phase state of ARF3 to enhance rice resistance to bacterial pathogens. Mol Plant. 2025;18:114–29. 10.1016/j.molp.2024.12.005. 39659014

[37] Qin T, Zhao H, Cui P, Albesher N, Xiong L. A nucleus-localized long non-coding rna enhances drought and salt stress tolerance. Plant Physiol. 2017;175:1321–36. 10.1104/pp.17.00574. PMC5664461 28887353

[38] Zhang P, He R, Yang J, Cai J, Qu Z, Yang R, et al. The long non-coding RNA DANA2 positively regulates drought tolerance by recruiting ERF84 to promote JMJ29-mediated histone demethylation. Mol Plant. 2023;16:1339–53. 10.1016/j.molp.2023.08.001. 37553833

[39] Cai J, Zhang Y, He R, Jiang L, Qu Z, Gu J, et al. LncRNA DANA1 promotes drought tolerance and histone deacetylation of drought responsive genes in *Arabidopsis*. EMBO Rep. 2024;25:796–812. 10.1038/s44319-023-00030-4. PMC10897447 38177920

[40] Zhang Y, Wang S, Li W, Wang S, Hao L, Xu C, et al. A long noncoding RNA HILinc1 enhances pear thermotolerance by stabilizing PbHILT1 transcripts through complementary base pairing. Commun Biol. 2022;5:1134. 10.1038/s42003-022-04010-7. PMC9606298 36289367

[41] He X, Guo S, Wang Y, Wang L, Shu S, Sun J. Systematic identification and analysis of heat-stress-responsive lncRNAs, circRNAs and miRNAs with associated co-expression and ceRNA networks in cucumber (*Cucumis sativus* L.). Physiol Plant. 2020;168:736–54. 10.1111/ppl.12997. 31125116

[42] Zhang Z, Zhong H, Nan B, Xiao B. Global identification and integrated analysis of heat-responsive long non-coding RNAs in contrasting rice cultivars. Theor Appl Genet. 2022;135:833–52. 10.1007/s00122-021-04001-y. 34846546

[43] Wang L, Zhu Y, Zhao M, Liu D, Liao C, Zhang H, et al. Genome-wide analysis of lncRNA in wheat (Triticum aestivum) and functional characterization of TalncR9 in response to drought stress. Front Plant Sci. 2025;16:1647354. 10.3389/fpls.2025.1647354. PMC12443737 40978798

[44] Zhang Z, Zhang R, Meng F, Chen Y, Wang W, Yang K, et al. A comprehensive atlas of long non-coding RNAs provides insight into grain development in wheat. Seed Biol. 2023;2:12. 10.48130/SeedBio-2023-0012.

[45] Madhawan A, Sharma A, Bhandawat A, Rahim MS, Kumar P, Mishra A, et al. Identification and characterization of long non-coding RNAs regulating resistant starch biosynthesis in bread wheat (*Triticum aestivum* L.). Genomics. 2020;112:3065–74. 10.1016/j.ygeno.2020.05.014. 32447006

[46] Lu Q, Xu Q, Guo F, Lv Y, Song C, Feng M, et al. Identification and characterization of long non-coding RNAs as competing endogenous RNAs in the cold stress response of *Triticum aestivum*. Plant Biol. 2020;22:635–45. 10.1111/plb.13119. 32249495

[47] Babaei S, Bhalla PL, Singh MB. Identifying long non-coding RNAs involved in heat stress response during wheat pollen development. Front Plant Sci. 2024;15:1344928. 10.3389/fpls.2024.1344928. PMC10876783 38379952

[48] Wang D, Zhang X, Cao Y, Batool A, Xu Y, Qiao Y, et al. TabHLH27 orchestrates root growth and drought tolerance to enhance water use efficiency in wheat. J Integr Plant Biol. 2024;66:1295–312. 10.1111/jipb.13670. 38695649

[49] Kumar S, Singh VP, Saini DK, Sharma H, Saripalli G, Kumar S, et al. Meta-QTLs, ortho-MQTLs, and candidate genes for thermotolerance in wheat (*Triticum aestivum* L.). Mol Breed. 2021;41:69. 10.1007/s11032-021-01264-7. PMC10236124 37309361

[50] Li L, Peng Z, Mao X, Wang J, Chang X, Reynolds M, et al. Genome-wide association study reveals genomic regions controlling root and shoot traits at late growth stages in wheat. Ann Bot. 2019;124:993–1006. 10.1093/aob/mcz041. PMC6881226 31329816

[51] Li A, Hao C, Wang Z, Geng S, Jia M, Wang F, et al. Wheat breeding history reveals synergistic selection of pleiotropic genomic sites for plant architecture and grain yield. Mol Plant. 2022;15:504–19. 10.1016/j.molp.2022.01.004. 35026438

[52] Wang H, Sun F, Shi Z, Yang Y, Ding Y, Zhang T, et al. A forward genetics strategy for high-throughput gene identification via precise image-based phenotyping of an indexed EMS mutant library. Adv Sci (Weinh). 2025;12:e14793. 10.1002/advs.202514793. PMC12713079 41017632

[53] Babaei S, Bhalla PL, Singh MB. Identifying long non-coding RNAs involved in heat stress response during wheat pollen development. Front Plant Sci. 2024;15:1344928. 10.3389/fpls.2024.1344928. PMC10876783 38379952

[54] Dai X, Zhuang Z, Zhao PX. PsRNATarget: a plant small RNA target analysis server (2017 release). Nucleic Acids Res. 2017;46:W49-54. 10.1093/nar/gky316. PMC6030838 29718424

[55] Shi X, Cui F, Han X, He Y, Zhao L, Zhang N, et al. Comparative genomic and transcriptomic analyses uncover the molecular basis of high nitrogen-use efficiency in the wheat cultivar Kenong 9204. Mol Plant. 2022;15:1440–56. 10.1016/j.molp.2022.07.008. 35864747

[56] Kim D, Paggi JM, Park C, Bennett C, Salzberg SL. Graph-based genome alignment and genotyping with HISAT2 and HISAT-genotype. Nat Biotechnol. 2019;37:907–15. 10.1038/s41587-019-0201-4. PMC7605509 31375807

[57] Li H, Handsaker B, Wysoker A, Fennell T, Ruan J, Homer N, et al. The sequence alignment/map format and SAMtools. Bioinformatics. 2009;25:2078–9. 10.1093/bioinformatics/btp352. PMC2723002 19505943

[58] Pertea M, Pertea GM, Antonescu CM, Chang T-C, Mendell JT, Salzberg SL. StringTie enables improved reconstruction of a transcriptome from RNA-seq reads. Nat Biotechnol. 2015;33:290–5. 10.1038/nbt.3122. PMC4643835 25690850

[59] Pertea G, Pertea M. GFF utilities: GffRead and GffCompare. F1000Res. 2020;9:ISCB Comm J-304. 10.12688/f1000research.23297.2. PMC7222033 32489650

[60] Kang YJ, Yang DC, Kong L, Hou M, Meng YQ, Wei L, et al. CPC2: a fast and accurate coding potential calculator based on sequence intrinsic features. Nucleic Acids Res. 2017;45:W12–6. 10.1093/nar/gkx428. PMC5793834 28521017

[61] Sun L, Luo H, Bu D, Zhao G, Yu K, Zhang C, et al. Utilizing sequence intrinsic composition to classify protein-coding and long non-coding transcripts. Nucleic Acids Res. 2013;41:e166. 10.1093/nar/gkt646. PMC3783192 23892401

[62] Li A, Zhou H, Xiong S, Li J, Mallik S, Fei R, et al. PLEKv2: predicting lncRNAs and mRNAs based on intrinsic sequence features and the coding-net model. BMC Genomics. 2024;25:756. 10.1186/s12864-024-10662-y. PMC11295476 39095710

[63] Langfelder P, Horvath S. WGCNA: an R package for weighted correlation network analysis. BMC Bioinformatics. 2008;9:559. 10.1186/1471-2105-9-559. PMC2631488 19114008

[64] Shannon P, Markiel A, Ozier O, Baliga NS, Wang JT, Ramage D, et al. Cytoscape: a software environment for integrated models of biomolecular interaction networks. Genome Res. 2003;13:2498–504. 10.1101/gr.1239303. PMC403769 14597658

[65] Schmittgen TD, Livak KJ. Analyzing real-time PCR data by the comparative C(T) method. Nat Protoc. 2008;3:1101–8. 10.1038/nprot.2008.73. 18546601

