# Supplementary material for: Identifying heat shock-responsive lncRNAs in wheat during grain filling
Source: BMC Plant Biol. 2026 Aug 25;26:1673. doi: 10.1186/s12870-026-09785-z (PMC13628808; doi:10.1186/s12870-026-09785-z)
Supplement: Supplementary file 1 [file 12870_2026_9785_MOESM1_ESM.pdf]

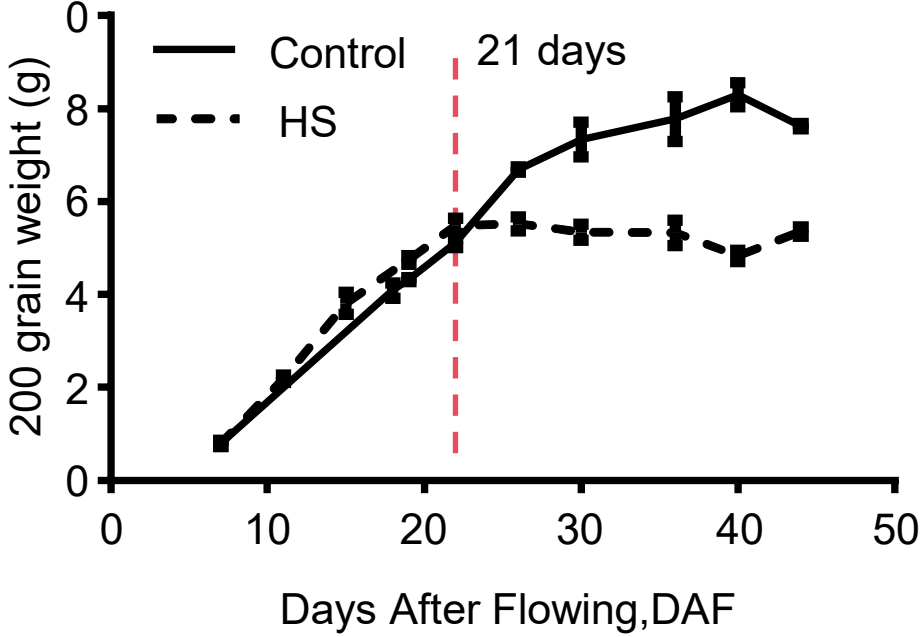
Control
HS
21 days
200 grain weight (g)
0
2
4
6
8
0
0
10
20
30
40
50
Days After Flowing,DAF
