# Supplementary material for: Identifying heat shock-responsive lncRNAs in wheat during grain filling
Source: BMC Plant Biol. 2026 Aug 25;26:1673. doi: 10.1186/s12870-026-09785-z (PMC13628808; doi:10.1186/s12870-026-09785-z)
Supplement: Supplementary file 2 [file 12870_2026_9785_MOESM2_ESM.pdf]

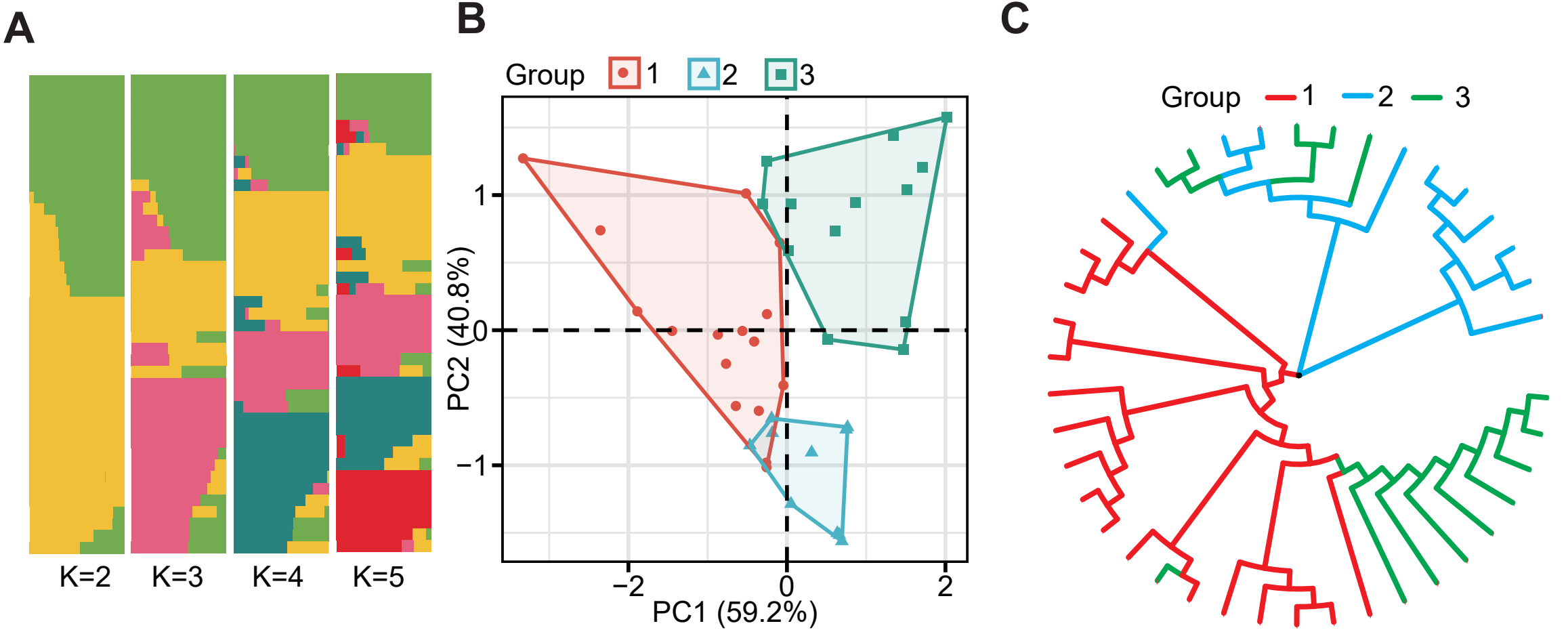

A
K=2
K=3
K=4
K=5
B
Group
1
2
3
1
0
−1
−2
0
2
PC1 (59.2%)
PC2 (40.8%)
C
Group
1
2
3
