# Supplementary material for: Identifying heat shock-responsive lncRNAs in wheat during grain filling
Source: BMC Plant Biol. 2026 Aug 25;26:1673. doi: 10.1186/s12870-026-09785-z (PMC13628808; doi:10.1186/s12870-026-09785-z)
Supplement: Supplementary file 3 [file 12870_2026_9785_MOESM3_ESM.pdf]

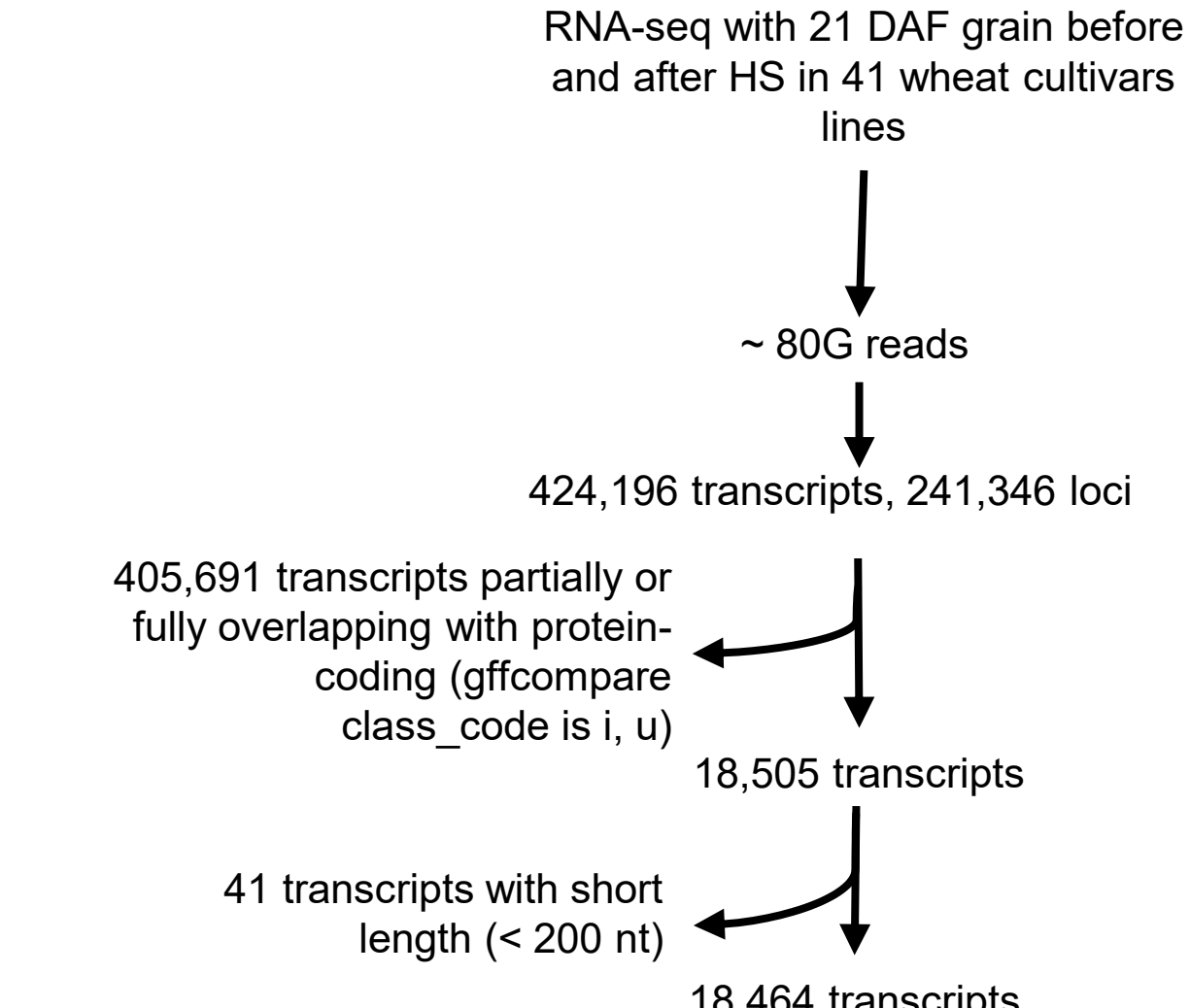
RNA-seq with 21 DAF grain before and after HS in 41 wheat cultivars lines
~ 80G reads
424,196 transcripts, 241,346 loci
405,691 transcripts partially or fully overlapping with protein-coding (gffcompare class_code is i, u)
18,505 transcripts
41 transcripts with short length (< 200 nt)
18,464 transcripts
9,000 transcripts with protein-coding potential (CPC2 score > 0.5, CNCI score >0, or PLEK score >0)
9,464 LncRNAs, 9,263 loci
