# Supplementary material for: Identifying heat shock-responsive lncRNAs in wheat during grain filling
Source: BMC Plant Biol. 2026 Aug 25;26:1673. doi: 10.1186/s12870-026-09785-z (PMC13628808; doi:10.1186/s12870-026-09785-z)
Supplement: Supplementary file 4 [file 12870_2026_9785_MOESM4_ESM.pdf]

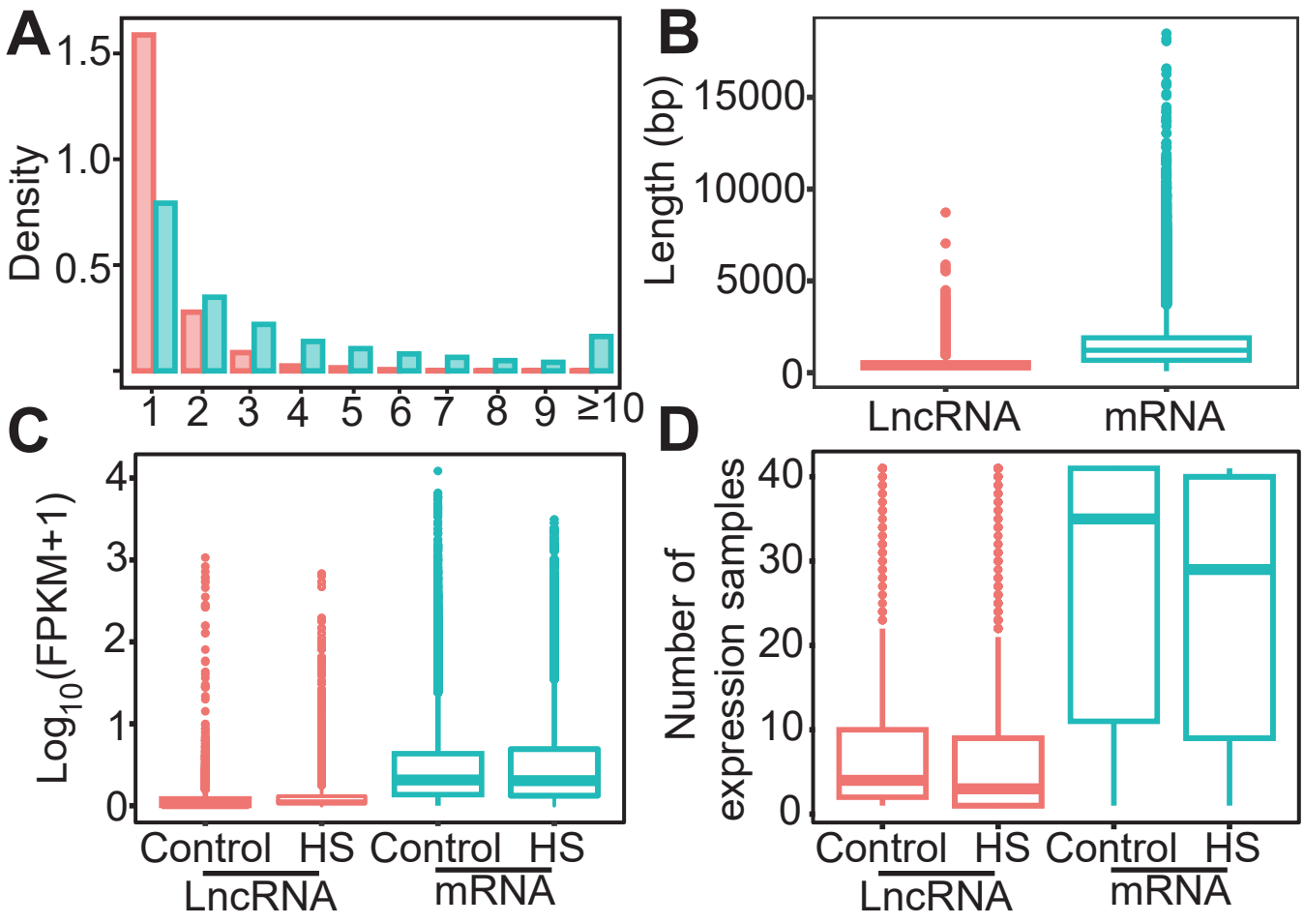
A
Density
1.5
1.0
0.5
1
2
3
4
5
6
7
8
9
≥10
B
Length (bp)
15000
10000
5000
0
LncRNA
mRNA
C
Log10(FPKM+1)
4
3
2
1
0
Control
HS
Control
HS
LncRNA
mRNA
D
Number of expression samples
40
30
20
10
0
Control
HS
Control
HS
LncRNA
mRNA
