# Supplementary material for: Identifying heat shock-responsive lncRNAs in wheat during grain filling
Source: BMC Plant Biol. 2026 Aug 25;26:1673. doi: 10.1186/s12870-026-09785-z (PMC13628808; doi:10.1186/s12870-026-09785-z)
Supplement: Supplementary file 5 [file 12870_2026_9785_MOESM5_ESM.pdf]

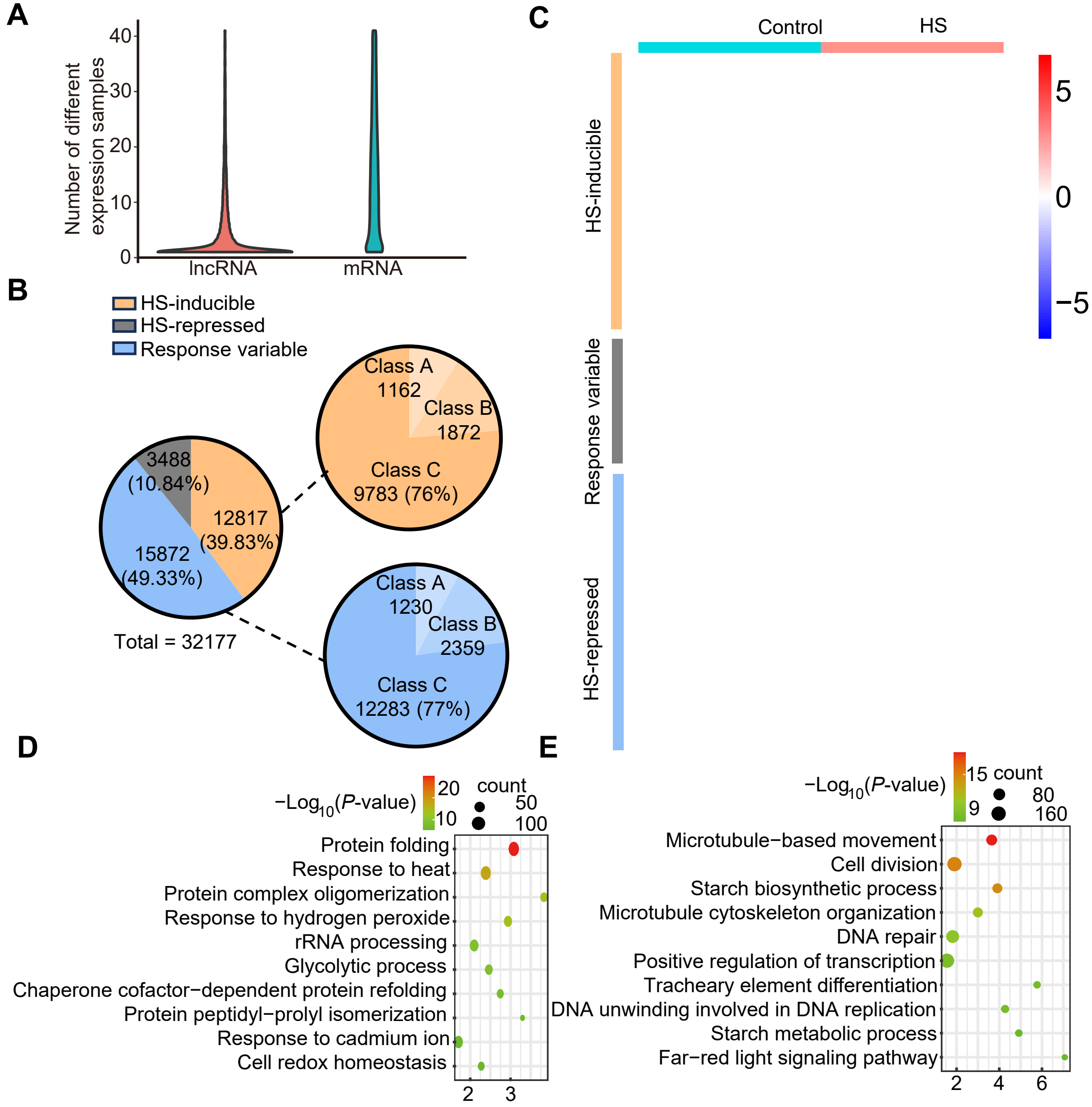
A
Number of different expression samples
40
30
20
10
0
lncRNA
mRNA
B
HS-inducible
HS-repressed
Response variable
3488 (10.84%)
12817 (39.83%)
15872 (49.33%)
Total = 32177
Class A 1162
Class B 1872
Class C 9783 (76%)
Class A 1230
Class B 2359
Class C 12283 (77%)
C
Control
HS
HS-inducible
Response variable
HS-repressed
5
0
−5
D
−Log10(P-value)
20
10
count
50
100
Protein folding
Response to heat
Protein complex oligomerization
Response to hydrogen peroxide
rRNA processing
Glycolytic process
Chaperone cofactor−dependent protein refolding
Protein peptidyl−prolyl isomerization
Response to cadmium ion
Cell redox homeostasis
2
3
E
−Log10(P-value)
15
9
count
80
160
Microtubule−based movement
Cell division
Starch biosynthetic process
Microtubule cytoskeleton organization
DNA repair
Positive regulation of transcription
Tracheary element differentiation
DNA unwinding involved in DNA replication
Starch metabolic process
Far−red light signaling pathway
2
4
6
